# Oral contraception following abortion: A systematic review and meta-analysis

**DOI:** 10.1097/MD.0000000000003825

**Published:** 2016-07-08

**Authors:** Yan Che, Xiaoting Liu, Bin Zhang, Linan Cheng

**Affiliations:** a Key Lab of Reproduction Regulation of NPFPC, Shanghai Institute of Planned Parenthood Research (SIPPR), WHO Collaborating Centre for Research in Human Reproduction, Shanghai; b Library and Institute of Medical Information,CAMS & PUMC, Beijing, China.

**Keywords:** combined oral contraceptives, contraceptives, induced abortion, medical abortion, surgical abortion, systematic review, vaginal bleeding

## Abstract

Oral contraceptives (OCs) following induced abortion offer a reliable method to avoid repeated abortion. However, limited data exist supporting the effective use of OCs postabortion. We conducted this systematic review and meta-analysis in the present study reported immediate administration of OCs or combined OCs postabortion may reduce vaginal bleeding time and amount, shorten the menstruation recovery period, increase endometrial thickness 2 to 3 weeks after abortion, and reduce the risk of complications and unintended pregnancies.

A total of 8 major authorized Chinese and English databases were screened from January 1960 to November 2014. Randomized controlled trials in which patients had undergone medical or surgical abortions were included. Chinese studies that met the inclusion criteria were divided into 3 groups: administration of OC postmedical abortion (group I; n = 1712), administration of OC postsurgical abortion (group II; n = 8788), and administration of OC in combination with traditional Chinese medicine postsurgical abortion (group III; n = 19,707).

In total, 119 of 6160 publications were included in this analysis. Significant difference was observed in group I for vaginal bleeding time (*P* = 0.0001), the amount of vaginal bleeding (*P* = 0.03), and menstruation recovery period (*P* < 0.00001) compared with the control groups. Group II demonstrated a significant difference in vaginal bleeding time (*P* < 0.00001), the amount of vaginal bleeding (*P* = 0.0002), menstruation recovery period (*P* < 0.00001), and endometrial thickness at 2 (*P* = 0.003) and 3 (*P* < 0.00001) weeks postabortion compared with the control group. Similarly, a significant difference was observed in group III for reducing vaginal bleeding time (*P* < 0.00001) and the amount of vaginal bleeding (*P* < 0.00001), shortening the menstruation recovery period (*P* < 0.00001), and increasing endometrial thickness 2 and 3 weeks after surgical abortion (*P* < 0.00001, all).

Immediate administration of OCs postabortion may reduce vaginal bleeding time and amount, shorten the menstruation recovery period, increase endometrial thickness 2 to 3 weeks after abortion, and reduce the risk of complications and unintended pregnancies.

## Abstract 2

Supplemental Digital Content is available in the text

## 1 Introduction

Unsafe medical or surgical abortions are one of the major public health concerns acknowledged by the international scientific community. The World Health Organization (WHO) estimates that nearly 20 million unsafe abortions are performed annually worldwide under inadequate conditions, with 97% of them taking place in developing countries. In 2008, the global fatality rate was 220 deaths per 100,000 unsafe abortions.^[1,2]^ Unsafe abortions account for about 13% of all maternal deaths, approximating to 47,000 deaths annually.^[2]^ As reported by China's National Health and Family Commission, 6 to 10 million abortions have been performed in recent years, that is, about 700 to 1100 abortions an hour. Furthermore, several Chinese surveys have revealed that nearly 91% of pregnant teenagers opt for abortions or repeated abortions.^[3]^

Postabortion contraception and family planning services are associated with high acceptance and use of contraceptives. However, a majority of the women are deprived of these services, especially in countries where abortion is legally regulated.^[4]^ Women seek medical or surgical abortion as a means to overcome contraceptive failure. They often tend to oversee the grave consequences of medical and surgical abortion on health and well-being and continue to adopt these unsafe methods because of prejudices such as adverse effects (AEs) of modern contraceptives on fertility.^[5–8]^ New-age women rely on the use of either medications or surgical procedures for abortion.^[5]^ Women opting for abortion in the 1st trimester of pregnancy are recommended pharmacological agents such as mifepristone, prostaglandin, progesterone antagonist, methotrexate, and tamoxifen, which are considered as good as surgery.^[9]^ Although the use of these pharmacological agents in the 2nd trimester has considerably improved during the past decade, surgery is still considered to have a lower risk of AEs.^[10]^ Evidence suggests that birth control measures, such as pills or intrauterine devices (IUDs), can be initiated immediately after abortion.^[11–15]^

Oral contraceptives (OCs), commonly used postabortion, can be classified as combined oral contraceptives (COCs) and progestin-only contraceptives on the basis of the hormone components.^[16,17]^ The use of COCs is considered effective, but the potential risks of age-related diseases such as cardiovascular disease associated with their use must not be overlooked. In addition, COCs are associated with an increased risk of venous thromboembolism. Although important, venous thromboembolism is not of clinical significance in nonhospitalized Chinese and perhaps other Asian women because of its low incidence; therefore, COCs may be considered a better choice in these groups of patients.^[18]^

Postabortion contraception with counseling and provision of contraceptive methods must be considered an integral part of comprehensive abortion care to prevent future unplanned pregnancies and associated risks.^[11]^ In this regard, the WHO (WHO, Guide Practical Guide for Programme Managers, 1997) and the International Federation of Gynecology and Obstetrics recommend postabortion contraception as one of the strategies for the prevention of unsafe abortion and its consequences.^[11,19]^ In addition, few studies have demonstrated the significance of providing contraception immediately postabortion; data suggest that almost all contraceptive methods can be initiated immediately postabortion.^[11,15]^

The Guidance on the Family Planning Service after the Induced Abortion, published by the Family Planning Branch of Chinese Medical Association (2011), briefly states that women should take OCs immediately after abortion as one of the measures for future contraception and avoidance of abortion.^[20]^ However, contraceptive use postmedical or surgical abortion is a major health concern; furthermore, there are limited data on safe abortion care and its implications in current clinical practice. Therefore, it is imperative for clinicians and drug researchers to understand the effect of administering OC immediately after an induced abortion on vaginal bleeding time and amount and menstruation recovery period; endometrial restoration after an operation and unintended pregnancy; and reduction of abortion complications. In the lack of evidence consistently supporting the effective use of OC postabortion, we performed this systematic review and meta-analysis to estimate the effect of OC postabortion on vaginal bleeding time and amount, menstruation recovery period, endometrial thickness, associated complications, and unintended pregnancies.

## 2 Materials and methods

This systematic review and meta-analysis was conducted according to the Preferred Reporting Items for Systematic Reviews and Meta-Analyses guidelines.^[21]^ Ethical approval was not necessary as our study did not involve any patient intervention or information.

### 2.1 Data sources and searches

A search strategy used by the Cochrane Collaboration's Pregnancy and Childbirth Group was followed. Eligible trials were identified from monthly searches of the 8 major authorized Chinese and English databases, including PubMed, EMBASE, Cochrane Library, Web of Science, Chinese Biomedical Database, China National Knowledge Infrastructure, Wanfang Data, and VIP Database, from January 1960 to November 2014. The key words for retrieval of relevant articles included “oral contraceptive,” “combined oral contraceptive,” “induced abortion,” “medical abortion,” and “surgical abortion.” In addition, the Cochrane Controlled Trials Register and PubMed were also searched without language restrictions. The search strategy is described in Supplemental Digital Content 1. All the studies identified through the search strategy were assessed for inclusion in the analysis. RevMan version 5.2 (Cochrane Collaboration, Oxford, UK) was used to enter the data and check for data accuracy.

### 2.2 Study selection

The review included only randomized controlled trials (RCTs) in which the patients had undergone medical or surgical abortions. Intervening measures included administration of OC on the same day of abortion or administration of OC plus traditional Chinese medicine postabortion (study groups). The control group included women following nonmedical intervention contraceptive methods (such as condom or withdraw), women not receiving any medication (blank control), or women receiving placebo. Meeting abstracts without any detailed minutes, cohort studies, case–control studies, pseudorandom studies, research on curative effects of different contraceptives, studies involving OC administration after the 2nd day of induced abortion, and surgical abortion with a sample size <100 were excluded from the analysis.

### 2.3 Outcome measures

The analysis aimed to assess the effect of OC administration immediately after abortion on vaginal bleeding time and amount, endometrial thickness 2 to 3 weeks after abortion, menstruation recovery period (duration from the surgery to the 1st menstrual period), postoperative complications (pelvic cavity infection, intrauterine adhesion [IUA], cervical adhesion, and incomplete abortion), and unintended pregnancies (during the valid period of OC).

### 2.4 Data extraction and quality assessment

The quality of reporting of RCTs was assessed under masked conditions using the Jadad scoring or Oxford quality scoring system, according to which a score of 1 to 3 represents low-quality research and a score of 4 to 7 represents high-quality research (Table 1).^[22]^ The quality of the included research studies was evaluated from the following aspects: generation of random sequences; blind method; dropout and loss to follow-up; and allocation concealment. A document management software was used to screen the documents retrieved from the databases in order to avoid repetition. We designed a data extraction form that was used by 2 independent investigators who reviewed the studies for eligibility, extracted the data, and assessed the study quality based on inclusion and exclusion criteria. The data indicators extracted from research included patient numbers in the research and control groups, vaginal bleeding time and amount, menstruation recovery period, complications (including pelvic cavity infection, IUA, cervical adhesion, and incomplete abortion), and unintended pregnancies. Discrepancies observed between the data in different research studies were unified, discussed, and, if required, consulted with a 3rd party.

**Table 1:**
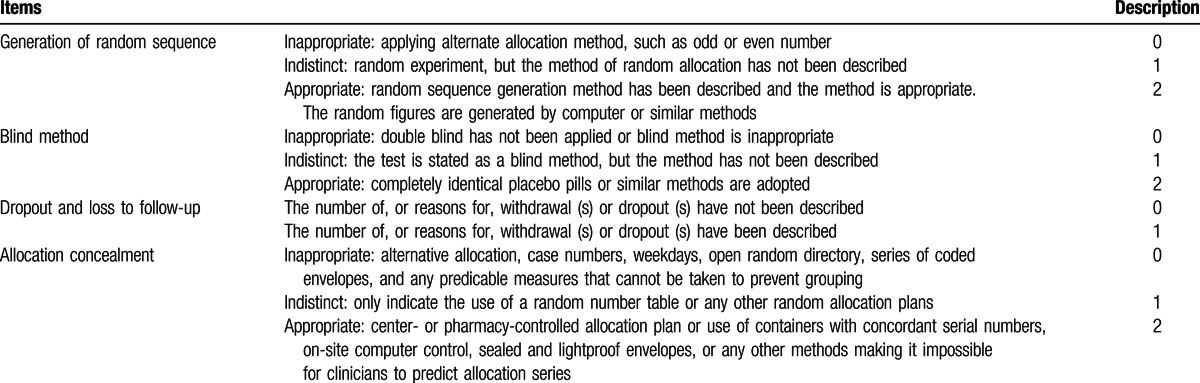
Jadad scale for randomized controlled studies.

### 2.5 Data synthesis and analysis

For dichotomous data, categorical variables were expressed by relative risk ratio (RR) with 95% confidence interval (CI), whereas continuous variables were expressed by mean difference (MD) and 95% CI. A heterogeneity test was conducted with Chi-square inspection, with the inspection level α = 0.1. The presence of statistical heterogeneity was determined using *I*^2^ statistics. As per the *Ι*^2^ estimate, when the degree of heterogeneity analysis index was found to be greater (*P* < 0.1, *Ι*^2^ > 50%), we used the random effects meta-analysis model to control confounding factors reducing the degree of heterogeneity, whereas when the analysis of indicators was found to have low heterogeneity (*P* ≥ 0.1, *I*^2^ < 50%) we applied the fixed effect model for combined analysis. Heterogeneity was considered substantial when *I*^2^ was >50%. A probability value <0.05 was considered statistically significant for the difference. The funnel plot technique was used to estimate the possibility of publication bias.

## 3 Results

### 3.1 Identified articles

**Figure 1:**
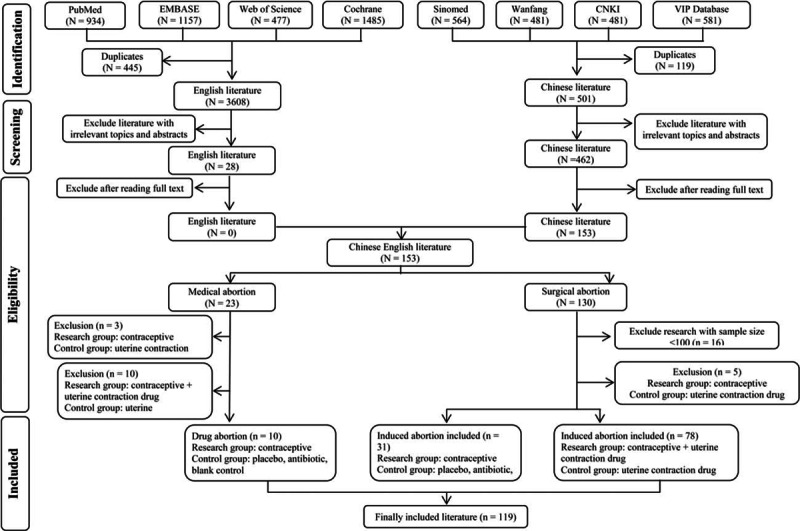
Flow chart of literature screening.

The search yielded 2107 Chinese articles and 4053 English articles. Articles with irrelevant topics and abstracts were excluded while screening. The eligibility of the articles for inclusion was tested after reading the full text, following which 28 English studies were excluded from the analysis, including studies comparing the therapeutic effect of different contraceptives (n = 11), studies with a sample size <100 (n = 9), studies with nonrandomized grouping methods or ineligible protocols (n = 5), and studies without a definite outcome measure (n = 3). Only 119 Chinese articles met the inclusion criteria. The selection process of the articles fulfilling the inclusion criteria for the meta-analysis is depicted in Fig. 1. The characteristics of the included studies are detailed in Table 2 .^[23–141]^ The included studies (n = 119) were classified into 2 categories: those discussing medical abortion and those discussing surgical abortion. A total of 10 research studies included OC administration immediately after medical abortion as the research or treatment group and administration of placebo, blank control, or other contraceptive measures as the control group (group I). In total, there were 109 studies on OC administration postsurgical abortion: 31 studies reported OC intake as the research group and placebo/microbiotic intake as the control group (group II) and 78 studies reported OC plus traditional Chinese medicines postsurgical abortion as the research group (group III). The methodological quality of the included studies as assessed by the Jadad scoring system is described in Table 3 .^[22,23–141]^

**Table 2:**
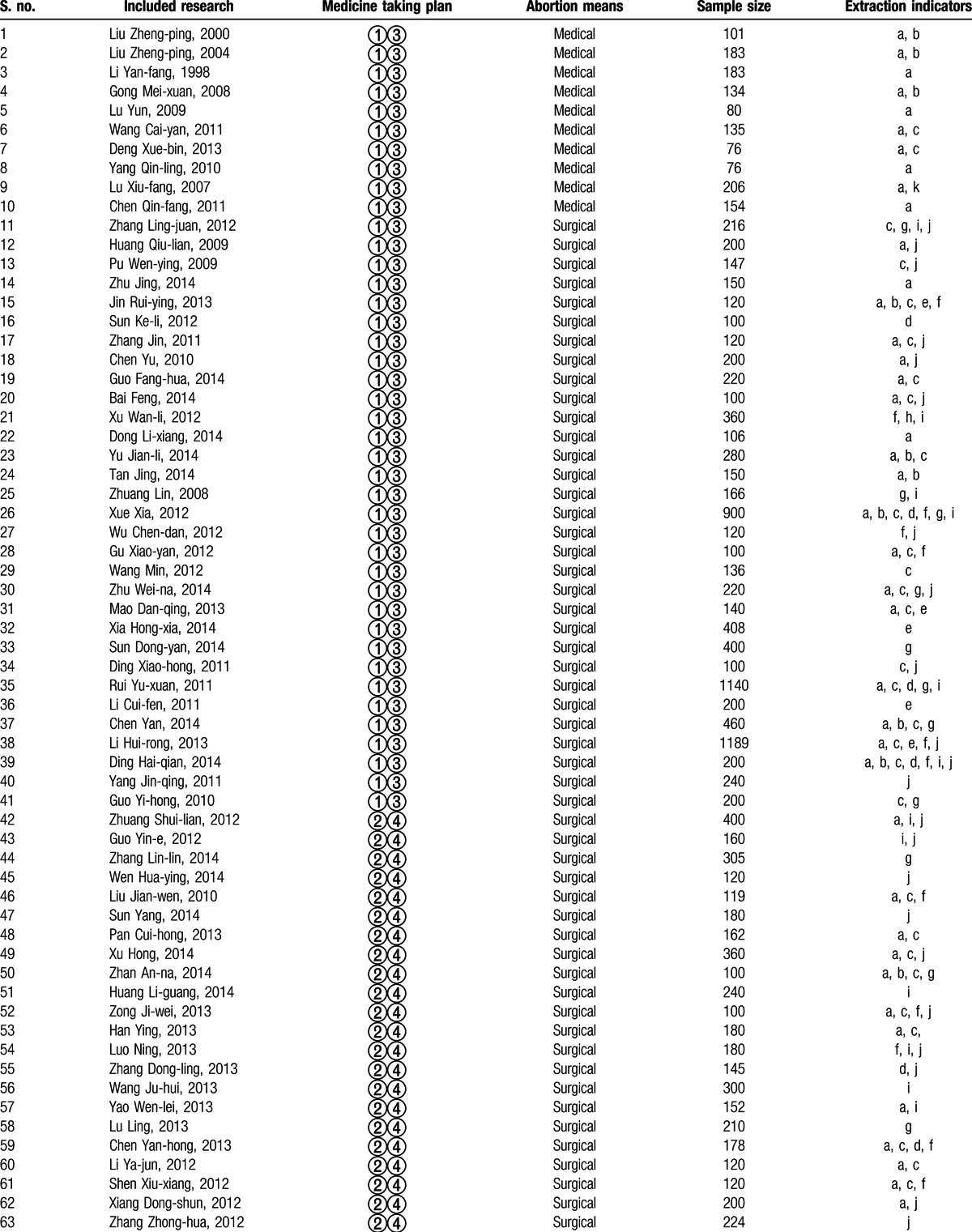
Essential features of the included researches^[23–141]^.

**Table 2 (Continued):**
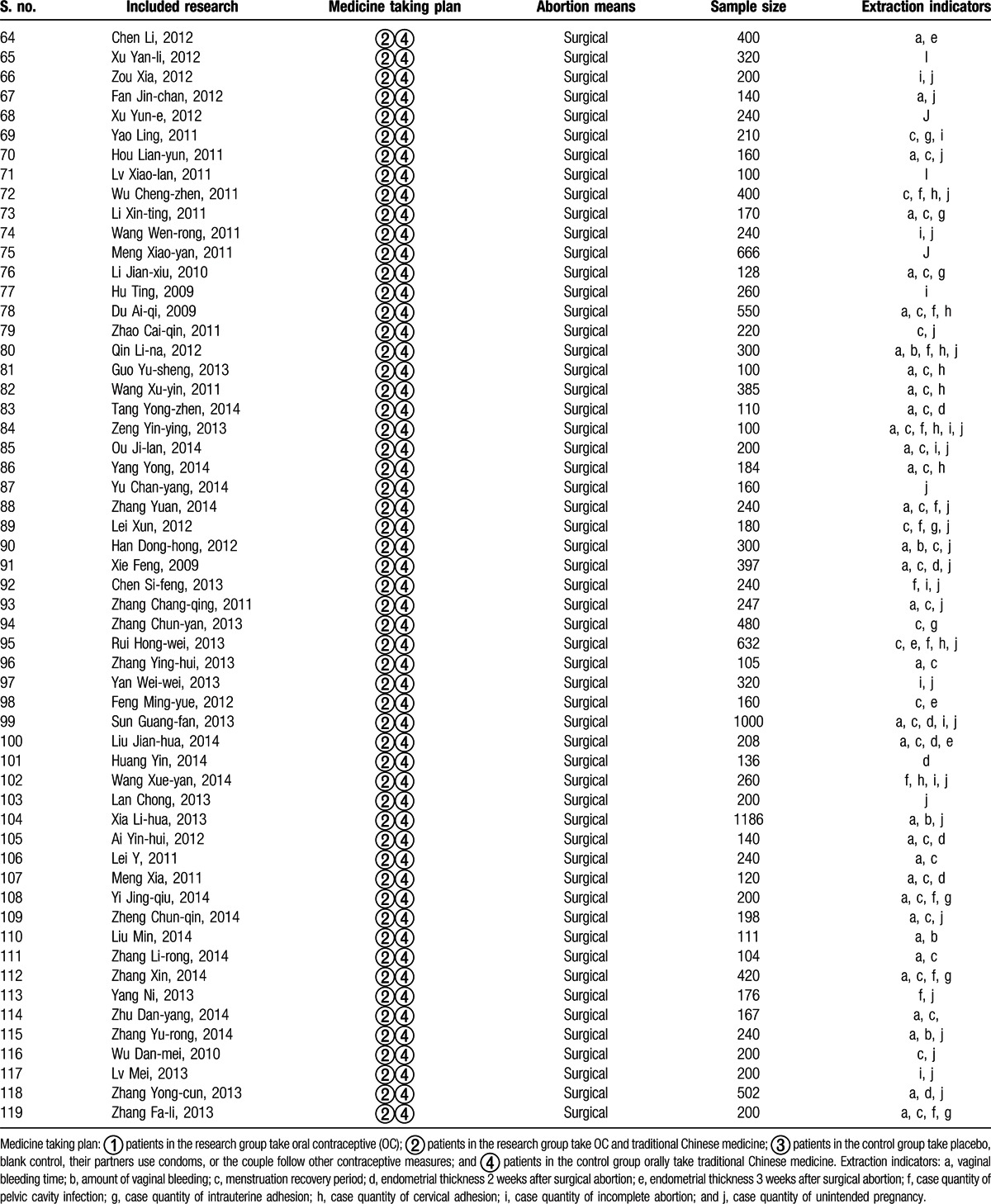
Essential features of the included researches^[23–141]^.

**Table 3:**
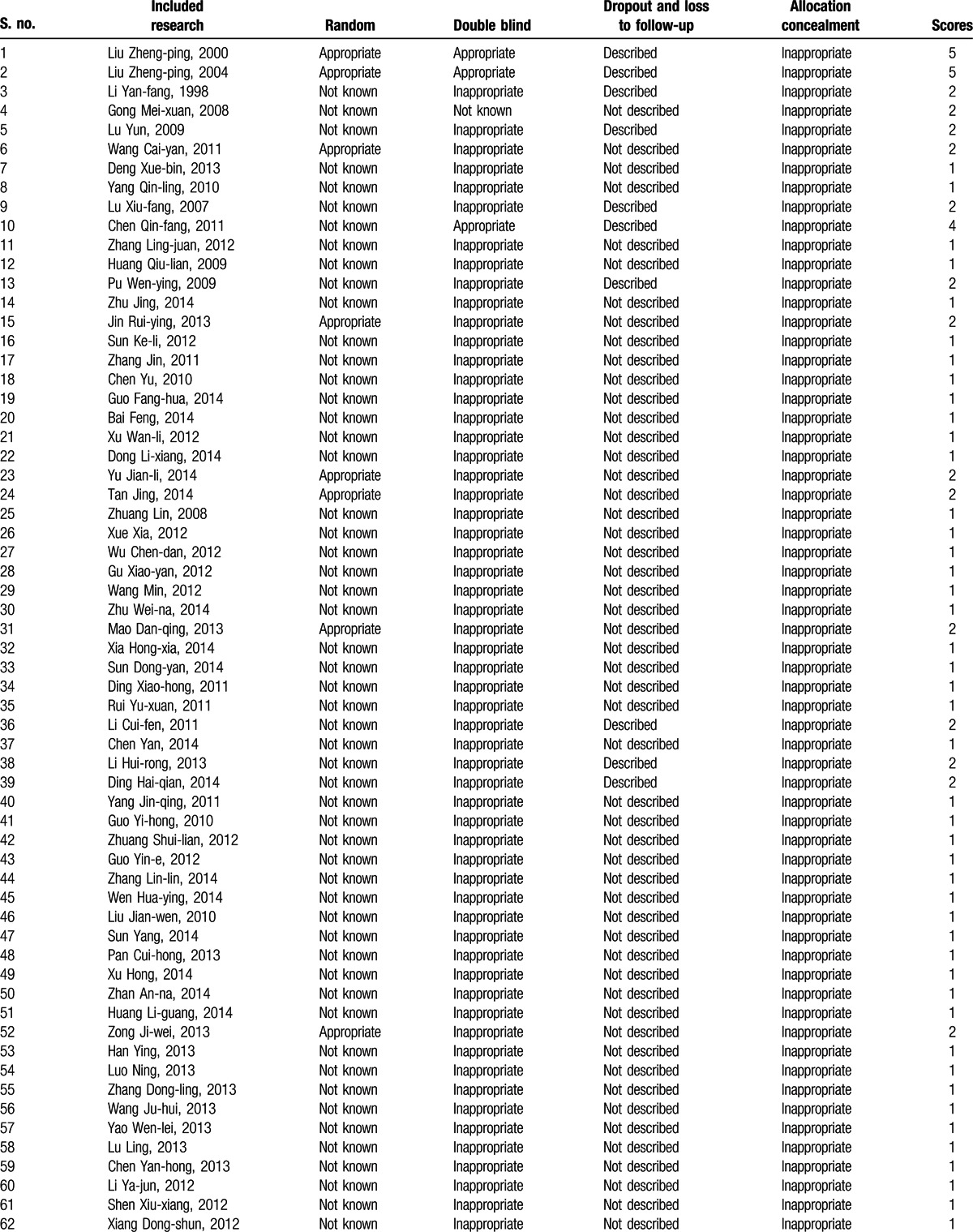
Evaluation of the methodological quality of the included research^[23–141]^.

**Table 3 (Continued):**
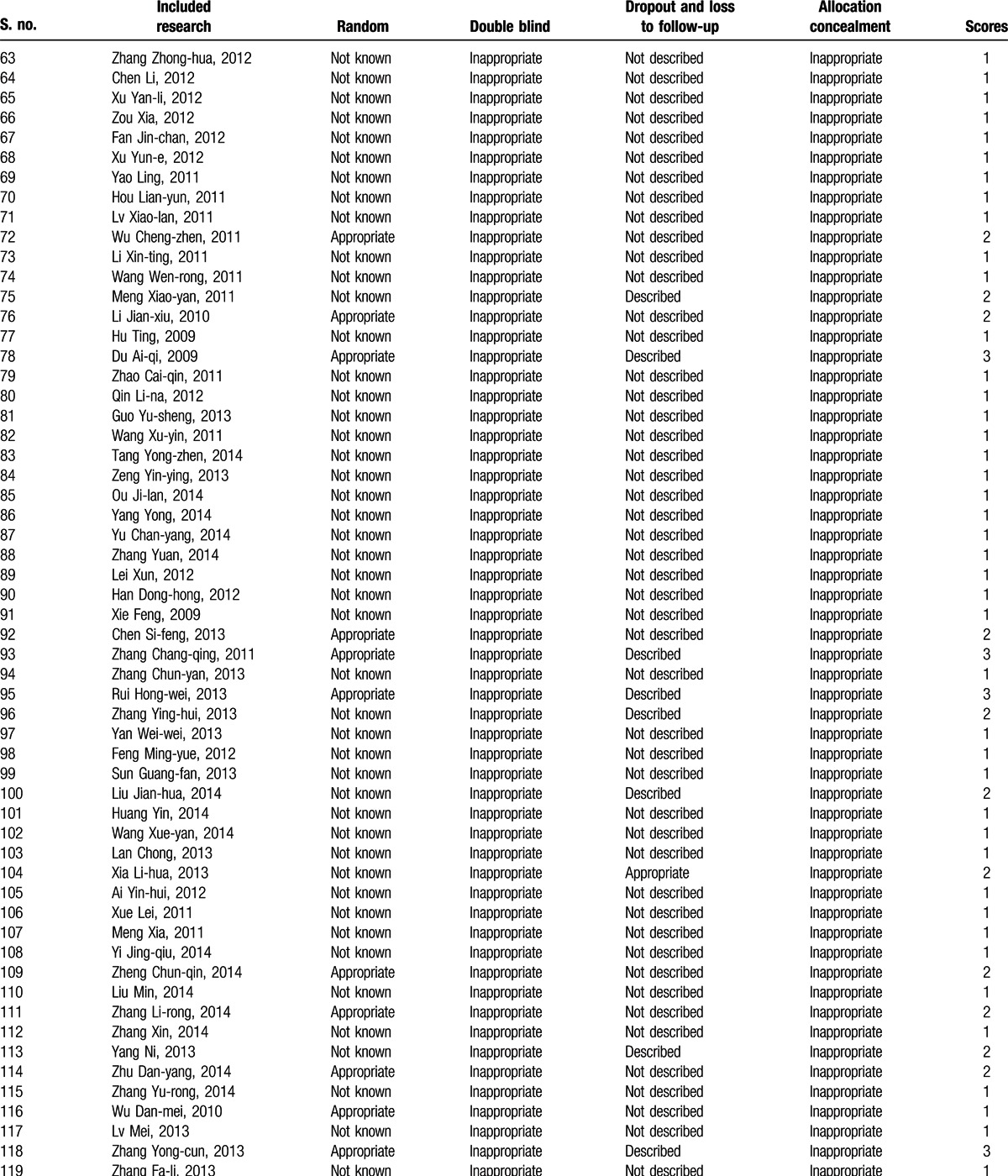
Evaluation of the methodological quality of the included research^[23–141]^.

### 3.2 OC administration immediately after medical abortion (group I)

Group 1 included a total of 10 studies with 1712 patients (817 patients in the treatment group and 895 patients in the control group; Table 4).

**Table 4:**
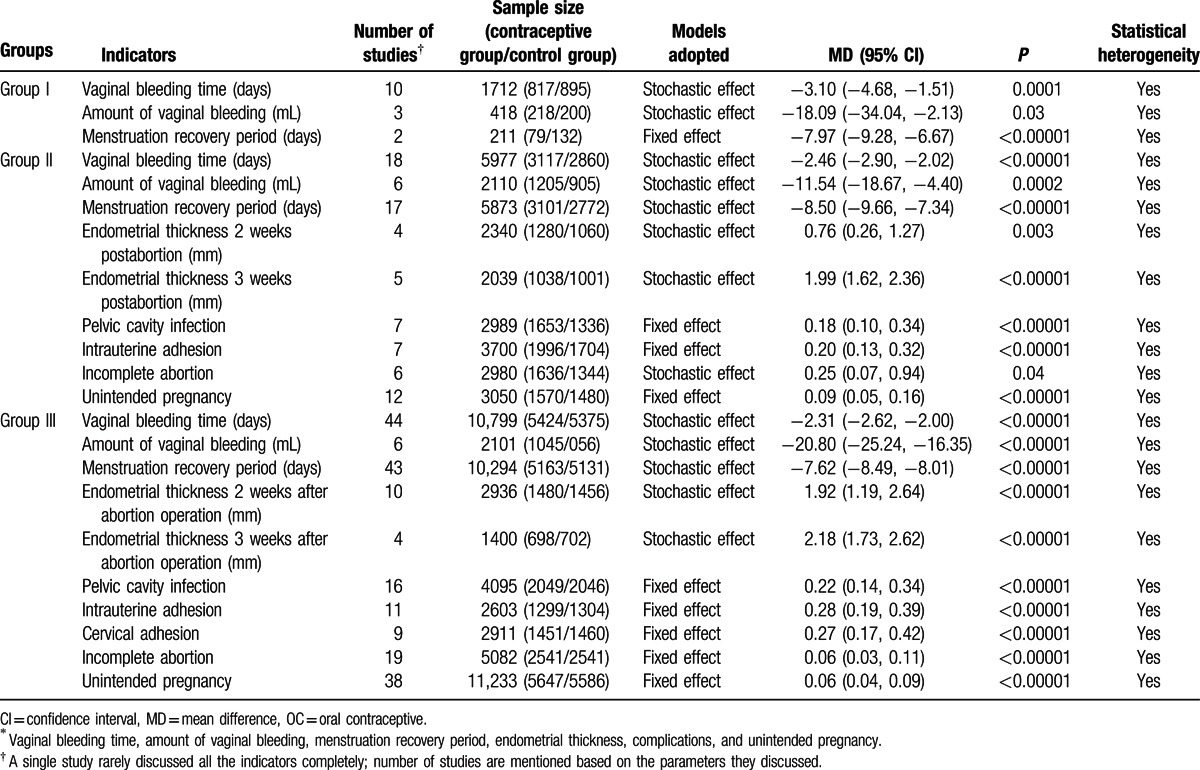
Effect of administering OCs postabortion on several variables^∗^.

#### 3.2.1 Effect on vaginal bleeding time and amount and menstruation recovery period

All of these studies discussed vaginal bleeding time (n = 10), whereas only a few studies discussed the amount of vaginal bleeding (n = 3), menstruation recovery period (n = 2), and unintended pregnancy (n = 1). A random effect model was applied for meta-analysis in studies discussing vaginal bleeding time and amount because of high heterogeneity, whereas a fixed effect model was applied for studies discussing menstruation period because of low heterogeneity (Supplemental Digital Content 2).^[23–141]^

**Figure 2:**
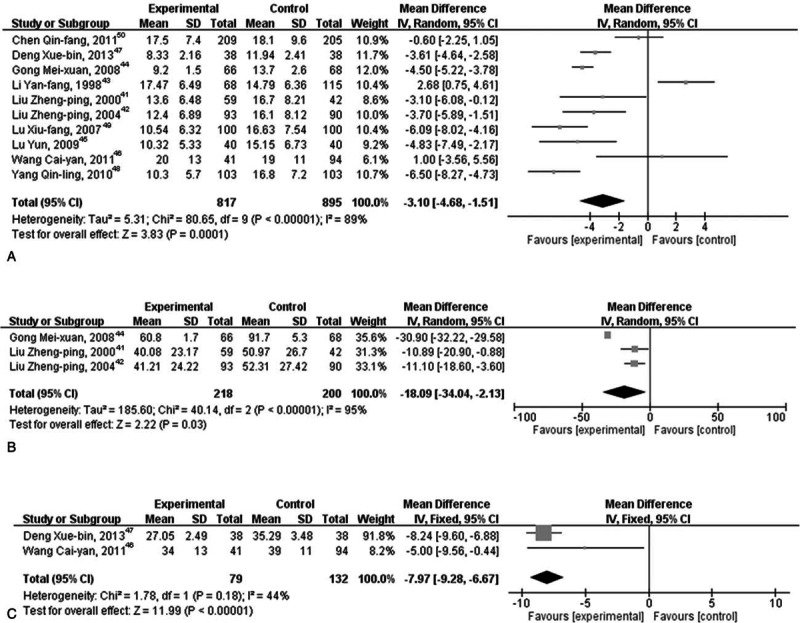
Effect of oral contraceptive (OC) immediately after medical abortion on vaginal bleeding time, amount of vaginal bleeding, and menstruation recovery period. (A) Effect of OC immediately after medical abortion on vaginal bleeding time, (B) effect of OC immediately after medical abortion on the amount of vaginal bleeding, and (C) effect of OC immediately after medical abortion on menstruation recovery period.

Group I patients demonstrated high heterogeneity among test results for vaginal bleeding time (χ^2^: 80.65, *I*^2^ = 89%; *P* < 0.00001). The effect of OCs in the treatment versus control group as depicted by a random effect model (Z = 3.83) indicated that OC administration demonstrated immediate effect in the treatment group after medical abortion compared with the control group (10 trials; MD: −3.10 days; 95% CI: −4.68 to −1.51; *P* = 0.0001; Fig. 2A). For the amount of vaginal bleeding, significantly high heterogeneity (χ^2^: 40.14 and *I*^2^ = 95%; *P* < 0.00001) was observed between the studies. The results suggested that there exists a significant difference (MD: −18.09 mL; 95% CI: −34.04 to −2.13; *P* = 0.03; Fig. 2B) between the treatment and control groups (Z = 2.22 and *P* *=* 0.03). For menstruation recovery, the test for overall effect between the treatment and control groups (Z = 11.99 and *P* < 0.00001) demonstrated a significant difference (MD: −7.97 days; 95% CI: −9.28 to −6.67; *P* < 0.00001) (Fig. 2C). It is evident that OC administration immediately after medical abortion has a significant effect in reducing vaginal bleeding time and amount, as well as menstruation recovery period.

### 3.3 OC administration immediately postsurgical abortion (group II)

Group II included 31 studies with 8788 patients (4552 patients in the treatment group and 4236 patients in the control group).

#### 3.3.1 Effect on vaginal bleeding time and amount, menstruation recovery period, and endometrial thickness

A total of 50 studies discussed vaginal bleeding time (n = 18), the amount of vaginal bleeding (n = 6), menstruation recovery period (n = 17), endometrial thickness 2 weeks postabortion (n = 4), and endometrial thickness 3 weeks postabortion (n = 5). A high heterogeneity was found to exist among all the studies and a random effect model was applied for meta-analysis.

**Figure 3:**
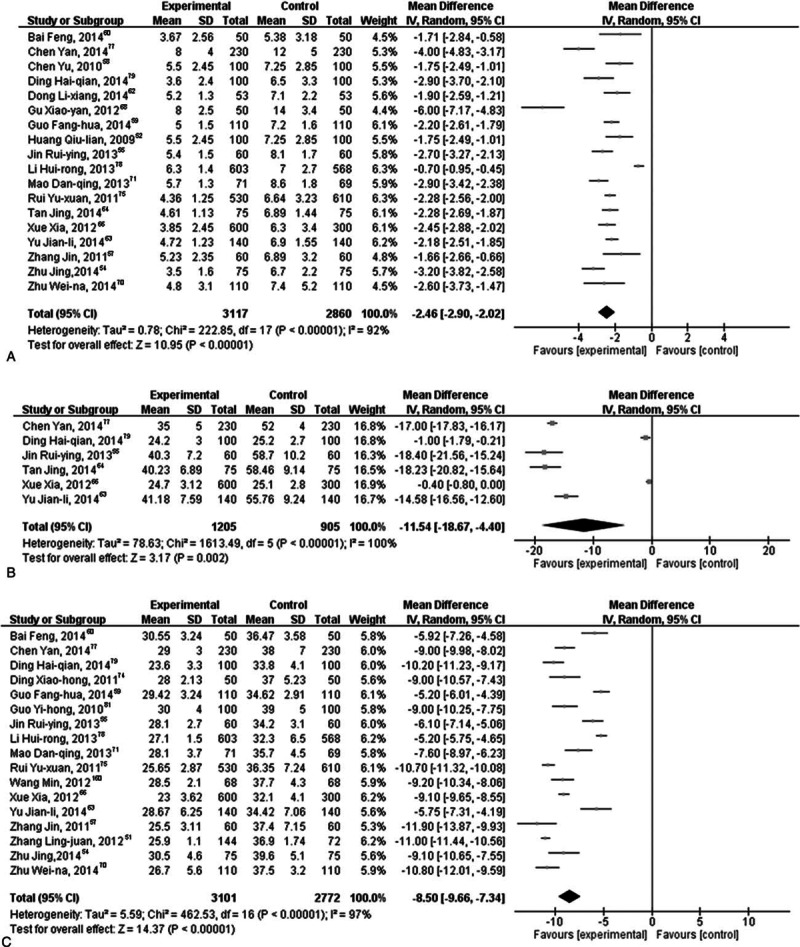
Effect of oral contraceptive (OC) immediately after surgical abortion on vaginal bleeding time, amount of vaginal bleeding, and menstruation recovery period. (A) Effect of OC immediately after surgical abortion on vaginal bleeding time, (B) effect of OC immediately after surgical abortion on the amount of vaginal bleeding, and (C) effect of OC immediately after surgical abortion on menstruation recovery period.

The effect of OCs in the treatment versus control group as depicted by a random effect model (Z = 10.95 and *P* < 0.00001) indicated that OC administration demonstrated an immediate effect in reducing vaginal bleeding time in the treatment versus control group after surgical abortion (18 trials; MD: −2.46 days; 95% CI: −2.90 to −2.02; *P* < 0.00001; Fig. 3A). The result showed a statistically significant difference (6 trials; MD: −11.54 mL; 95% CI: −18.67 to −4.40; *P* = 0.0002; Fig. 3B) between the treatment versus control group (Z = 3.17 and *P* = 0.002) in reducing the amount of vaginal bleeding postsurgical abortion. The overall effect (Z = 14.37 and *P* = l.00001) demonstrated a significant difference between the treatment versus control group (17 trials; MD: −8.50 days; 95% CI: −9.66 to −7.34; *P* < 0.00001) in reducing the menstruation recovery period, indicating the effectiveness of OC administration immediately postsurgical abortion (Fig. 3C). We observed a statistical difference in endometrial thickness 2 weeks postabortion (MD, 0.76 mm; 95% CI, 0.26, 1.27; *P* = 0.003) and 3 weeks postabortion (MD, 1.99 mm; 95% CI, 1.62, 2.36; *P* < 0.00001) in the treatment group compared with the control group.

#### 3.3.2 Effect on the occurrence of complications and unintended pregnancy

Of the included studies, 7 discussed pelvic cavity infection, 7 discussed IUA, 1 research discussed cervical adhesion, 6 studies discussed incomplete abortion, and 12 studies discussed unintended pregnancy. Because of the low heterogeneity, a fixed effect model was applied for meta-analysis in studies discussing pelvic cavity infection, IUA, and unintended pregnancy. A statistically significant difference was observed between the treatment and control groups in pelvic cavity infection (RR, 0.18; 95% CI, 0.10, 0.34; *P* < 0.00001), IUA (RR, 0.20; 95% CI, 0.13, 0.32; *P* < 0.00001), incomplete abortion (RR, 0.25; 95% CI, 0.07, 0.94; *P* = 0.04), and unintended pregnancy (RR, 0.09; 95% CI, 0.05, 0.16; *P* < 0.00001; Supplemental Digital Content 3).^[23–141]^

### 3.4 Administration of OCs and traditional Chinese medicine immediately after surgical abortion (group III)

Group III included 78 studies with 19,707 patients (9865 participants in the treatment group and 9842 participants in the control group). The participants in the treatment group received both OC and traditional Chinese medicine immediately after surgical abortion, whereas the participants in the control group received only traditional Chinese medicine.

#### 3.4.1 Effect on vaginal bleeding time and amount, menstruation period, and endometrial thickness

In group III, a total of 44 studies discussed vaginal bleeding time, 6 studies discussed the amount of vaginal bleeding, 43 studies discussed menstruation recovery period, 10 studies discussed endometrial thickness 2 weeks postsurgical abortion, and 4 studies discussed endometrial thickness 3 weeks postsurgical abortion. A random effect model was applied for meta-analysis because of the high heterogeneity observed in all the studies, except for those discussing endometrial thickness 3 weeks postsurgical abortion.

**Figure 4:**
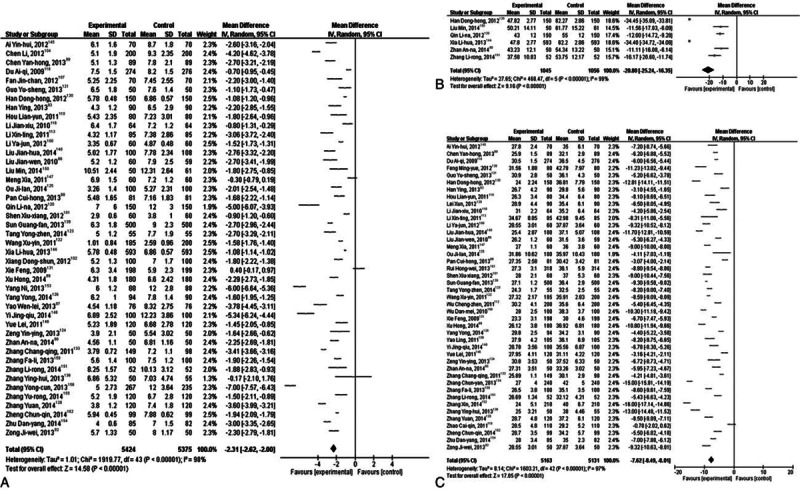
Effect of oral contraceptive (OC) and traditional Chinese medicine immediately after surgical abortion on vaginal bleeding time, amount of vaginal bleeding, and menstruation recovery period. (A) Effect of OC and traditional Chinese medicine immediately after surgical abortion on vaginal bleeding time, (B) effect of OC and traditional Chinese medicine immediately after surgical abortion on the amount of vaginal bleeding, and (C) effect of OC and traditional Chinese medicine immediately after surgical abortion on menstruation recovery period.

The random effect model (Z = 14.58 and *P* < 0.00001) indicated that OC administration with traditional Chinese medicine demonstrated immediate effect in the treatment group in reducing vaginal bleeding time after surgical abortion compared with the control group (44 trials; MD: −2.31; 95% CI: −2.62 to −2.00; *P* < 0.00001; Fig. 4A). The results suggested that there exists a significant difference (6 trials; MD: −20.80; 95% CI: −25.24 to −16.35; *P* < 0.00001; Fig. 4B) between the treatment and control groups (Z = 9.16 and *P* = 0.00001) in reducing the amount of vaginal bleeding postsurgical abortion. The test for overall effect between the treatment and control groups (Z = 17.05 and *P* < 0.00001) demonstrated a significant difference (43 trials; MD: −7.62; 95% CI: −8.49 to −8.01; *P* < 0.00001) in reducing menstruation recovery period postsurgical abortion (Fig. 4C).

A statistically significant difference was observed in the increase in endometrial thickness 2 weeks after abortion (MD, 1.92 mm; 95% CI, 1.19, 2.64; *P* < 0.00001) and 3 weeks after abortion (MD, 2.18 mm; 95% CI, 1.73, 2.62; *P* < 0.00001) between the treatment and control groups.

#### 3.4.2 Effect on the occurrence of complications and unintended pregnancy

In group III, a total of 16 studies discussed pelvic cavity infection, 11 studies discussed IUA, 9 research studies discussed cervical adhesion, 19 studies discussed incomplete abortion, and 38 studies discussed unintended pregnancy. A fixed effect model was applied for analysis because of the presence of low heterogeneity in all the studies with respect to pelvic cavity infection, IUA, cervical adhesion, incomplete abortion, and unintended pregnancy. A statistically significant difference was observed in the reduction of pelvic cavity infection (RR, 0.22; 95% CI, 0.14, 0.34; *P* < 0.00001), IUA (RR, 0.28; 95% CI, 0.19, 0.39; *P* < 0.00001), cervical adhesion (RR, 0.27; 95% CI, 0.17, 0.42; *P* < 0.00001), incomplete abortion (RR, 0.06; 95% CI, 0.03, 0.11; *P* < 0.00001), and unintended pregnancy (RR, 0.06; 95% CI, 0.04, 0.09; *P* < 0.00001) in the treatment group compared with the control group.

### 3.5 Assessment of publication bias

**Figure 5:**
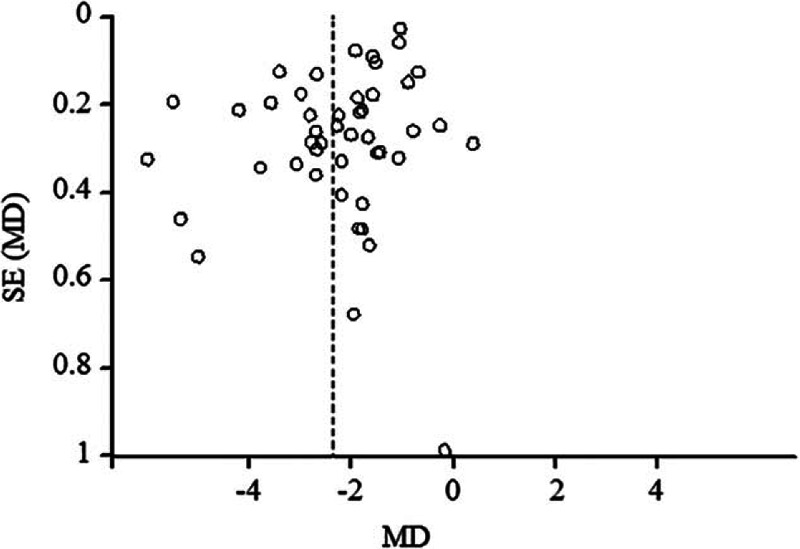
Funnel plot of publication bias on oral contraceptives (OCs) and traditional Chinese medicine after surgical abortion on vaginal bleeding time.

A funnel plot of the standard error of MD against MD demonstrated the possibility of publication bias in the studies among the 3 groups. Figure 5 shows the publication bias funnel plot for the effect of both OC and traditional Chinese medicine after surgical abortion on vaginal bleeding time.

## 4 Discussion

To the best of our knowledge, this is the 1st largest systematic review and meta-analysis that has covered a big time frame from January 1960 to November 2014 and assessed the effect of OC administration immediately postabortion on vaginal bleeding time and amount, menstruation recovery period, endometrial thickness, associated complications, and unintended pregnancies. Although several clinical trials have studied the effect of contraception postabortion, there still exists an unmet gap. Research seeking better clarity and definite conclusions for effective healthcare management in this regard is ongoing. Nonetheless, no study to date has provided cumulative data on the effect of OC administration postabortion for these variables.

Contraceptive counseling and provision of contraceptive methods are now considered an integral part of any abortion care. This aids in preventing future unplanned pregnancies, risks, complications, and subsequent abortions. As per Safe Abortion: Technical and Policy Guidance for Health Systems by the WHO, “Women may start hormonal contraception at the time of surgical abortion, or as early as the time of administration of the 1st pill of a medical abortion regimen.” Following medical abortion, an IUD may be inserted when it is reasonably certain that the woman is no longer pregnant (strength of recommendation: strong; quality of evidence based on RCTs: very low).^[142]^ Nevertheless, evidence suggests that almost all the contraceptive methods can be initiated immediately postabortion.^[11,15]^ In such a scenario, this study will provide comprehensive guidance to clinicians substantiating the use of immediate OC administration postabortion and its effect on various potential variables (i.e., vaginal bleeding time and amount, menstruation recovery duration, endometrial thickness, complications, and unintended pregnancies).

Several studies have been conducted to study the effect of contraceptives postabortion on vaginal bleeding time and amount and the duration of menstruation recovery.^[11,143–150]^ Vaginal bleeding, irrespective of medical or surgical abortion, is associated with significant morbidity and mortality rates and is regarded as a troublesome, unsolved concern. An estimate of blood loss due to vaginal bleeding is approximately >500 mL with transfusion or hospitalization, approximating to 40% of postabortion mortality, which is a major concern.^[143]^ Known etiologies include perforation, cervical laceration, retained tissue, abnormal placentation, uterine atony, and coagulopathy. Many strategies are used to prevent hemorrhage, with uterotonics being recommended as the primary treatment agent by the Society of Family Planning Guidelines 2012 and National Guideline Clearinghouse, United States.^[143,144]^ Nonetheless, many traditional Chinese medicines (such as Gong Xue Ning, Xuejie Jiawei, Bai Shao [white peony root], Xu Duan, teasel root, Shenqi Shenghua decoction, and Azalea flower) are reported to be effective in reducing the amount of vaginal bleeding and shortening vaginal bleeding time after medical abortion with minimal AEs.^[145–147]^ Our review provided the assessment of RCTs focusing on bleeding outcomes for prophylactic use of traditional Chinese medicine and COCs such as drospirenone, Marvelon, levonorgestrel, and ethinylestradiol. A statistically significant difference was found in vaginal bleeding time (MD, −3.10 days; 95% CI, −4.68, −1.51; *P* = 0.0001), the amount of vaginal bleeding (MD, −18.09 mL; 95% CI, −34.04, −2.13; *P* = 0.03), and menstruation recovery period (MD, −7.97 days; 95% CI, −9.28, −6.67; *P* < 0.00001) in COC administration after medical abortion and in COC administration after surgical abortion (vaginal bleeding time: MD, −2.46 days; 95% CI, −2.90, −2.02; *P* < 0.00001; the amount of vaginal bleeding: MD, −11.54 mL; 95% CI, −18.67, −4.40; *P* = 0.0002; and menstruation recovery period: MD, −8.50 days; 95% CI, −9.66, −7.34; *P* < 0.00001). In addition, our analysis on the administration of COC and traditional Chinese medicine demonstrated its efficacy in reducing vaginal bleeding time and amount and menstruation recovery period postsurgical abortion (vaginal bleeding time: MD, −2.37 days; 95% CI, −2.70, −2.05; *P* < 0.00001; amount of vaginal bleeding: MD, −20.80 mL; 95% CI, −25.24, −16.35; *P* < 0.00001; and menstruation recovery period: MD, −7.62 days; 95% CI, −8.49, −8.01; *P* < 0.00001).

Furthermore, endometrial thickness is a useful predictor of postabortion intrauterine pathology and acts as an indirect indicator of endometrial receptivity, which is associated with successful pregnancy outcomes. A positive correlation exists between endometrial thickness and pregnancy rates. A growing body of evidence suggests that positive pregnancy requires a minimum endometrial thickness of 4 to 6 mm, whereas a maximum endometrial thickness of 8 to 10 mm allows successful implantation and pregnancy. However, literature is scant and has reported conflicting results.^[151,152]^ Thus, endometrial thickness becomes an important parameter to be analyzed postabortion to address fertility concerns of women undergoing abortion. We analyzed 9 studies on OC administration immediately after surgical abortion and found that endometrial thickness 2 weeks (MD, 0.76 mm; 95% CI, 0.26, 1.27; *P* = 0.003) and 3 weeks postabortion (MD, 1.99 mm; 95% CI, 1.62, 2.36; *P* < 0.00001) had significantly increased in the treatment group than in the control group. Nonetheless, administration of OC and traditional Chinese medicine was analyzed in 14 studies, which revealed increasing endometrial thickness 2 weeks (MD, 1.92 mm; 95% CI, 1.19, 2.64; *P* < 0.00001) and 3 weeks after abortion (MD, 2.18 mm; 95% CI, 1.73, 2.62; *P* < 0.00001) compared with the control group.

Abortion poses few complications, including pelvic inflammatory disease, incomplete abortion, IUA, and unintended pregnancy. Pelvic inflammatory disease is the most commonly encountered serious infection postabortion, the risk of which is multiplied with each succeeding abortion as reported in the literature.^[153]^ It may further lead to infertility or ectopic pregnancy.^[154]^ Postabortion, acute symptoms of pelvic pain, fever, and bleeding may signify problems associated with the endometrium. Although antibiotics are recommended to control infection postabortion, selection becomes difficult and unclear because of the availability of a wide variety of pathogens.^[155]^ On the other hand, few studies have reported that protection against pelvic infection is one of the most important noncontraceptive benefits of OCs.^[156,157]^ As per the CDC Guidelines for Prevention and Management of Pelvic Inflammatory Disorder, OCs may reduce the risk of pelvic inflammatory disease that is not attributable to *Chlamydia trachomatis*,^[158]^ likely by thickening of mucus. Although mucus thickening actually prevents the entry of sperms, it may also prevent bacterial entry. Another possible complication is incomplete abortion, which is caused by unsuccessful evacuation or expulsion of the gestational sac, and can be determined by endometrial thickness. OCs are usually recommended for incomplete abortion possibly because of their uterine-inducing and cervix-softening abilities, resulting in complete evacuation of the uterus.^[11,15]^ IUA is a possible complication of surgical abortion, which can further result in pelvic pain, infection, infertility by tubal occlusion, distortions in the symmetry of the uterine cavity, damage to the basalis layer of the endometrium, menstrual disturbances, and premature labor.^[11,148–150]^ It thus becomes a topic of major concern postabortion. Westendorp et al^[159]^ reported that OC administration was associated with a relative risk of 0.55 for IUA development; however, the finding was nonsignificant (95% CI, 0.2–1.8). Data on the prevalence and treatment of IUDs are scarce. In this analysis, we found that OC administration postsurgical abortion resulted in statistical difference in pelvic cavity infection (RR, 0.18; 95% CI, 0.10, 0.34; *P* < 0.00001), IUA (RR, 0.20; 95% CI, 0.13, 0.32; *P* < 0.00001), incomplete abortion (RR, 0.25; 95% CI, 0.07, 0.94; *P* = 0.04), and unintended pregnancy (RR, 0.09; 95% CI, 0.05, 0.16; *P* < 0.00001). Studies on administration of OC plus traditional Chinese medicines reported reduced pelvic cavity infection (RR, 0.22; 95% CI, 0.14, 0.34; *P* < 0.00001), IUA (RR, 0.28; 95% CI, 0.19, 0.39; *P* < 0.00001), cervical adhesion (RR, 0.27; 95% CI, 0.17, 0.42; *P* < 0.00001), incomplete abortion (RR, 0.06; 95% CI, 0.03, 0.11; *P* < 0.00001), and unintended pregnancy (RR, 0.06; 95% CI, 0.04, 0.09; *P* < 0.00001) in the treatment group compared with the control group. Our analysis will help establish a strategy to provide optimal protection against these complications.

Overall, the present meta-analysis is robust, as it included a total of 119 Chinese articles by strictly scrutinizing a pool of 6160 articles retrieved from 8 major Chinese and English databases. The authors critically evaluated and statistically combined the results of comparable studies from a large pool of studies and presented the data with high statistical power and value-added estimates of the effect size of an intervention or an association. The results of the present analysis revealed that a statistically significant difference existed in aspects of reducing vaginal bleeding time and amount and menstruation recovery period; reducing both complications and unintended pregnancies; and increasing endometrial thickness 2 and 3 weeks after surgical abortion between taking OCs or both OC and traditional Chinese medicines immediately after surgical abortion or on the same day.

However, there are several important limitations in this analysis that do not allow definitive conclusions to be drawn. First, all the 119 studies included are RCTs with mostly low research quality (low-quality research/high-quality research: group I, 7/3; group II, 31/0; and group III, 78/0), indistinct randomization, blinding, inappropriate allocation concealment, and undescribed data of dropout patients and those lost to follow-up. Second, only Chinese articles were included and English articles were excluded, mostly because of small sample size, nonrandomized grouping, ineligible protocol, or indefinite outcome measure. This may impact the final meta-analysis. Third, although the authors have tried their best to present this systematic and quantitative synthesis of evidence with unbiased results by analyzing the complete information available from January 1960 to November 2014, the funnel plot asymmetry suggests a possible selection bias. Nonetheless, most of the articles included are published publicly and a possibility of missing out findings from gray literature may also contribute to publication bias. Furthermore, sensitivity analysis was not performed, which may impact the robustness of the final outcome. Last, variations in different potential influencing factors including contraceptives, dosing time, different surgical techniques and estimation methods, different gestational ages, and terms of pregnancy may have contributed to the high heterogeneity and may have biased the results in an unknown way. No adjustments were made for confounding variables such as prior induced abortion or time of contraception administration. Given the varying quality of studies included in our meta-analysis, the study quality itself could be responsible for the heterogeneity. In this regard, the result of this review should be interpreted with caution, as the overall quality of the included studies needs to be considered as poor to average.

## 5 Conclusion

The present study indicates that administration of OC alone or in combination with traditional Chinese medicines immediately postabortion may have an impact on reducing vaginal bleeding time and amount, as well as the menstruation recovery period, increasing endometrial thickness 2 to 3 weeks after abortion, and reducing complications and unintended pregnancies. However, because of the limitation in the quantity and quality of the included studies, the conclusion of this analysis should be further verified by larger samples and high-quality research studies.

## Supplementary Material

Supplemental Digital Content
