# Supplementary material for: Oral contraception following abortion: A systematic review and meta-analysis
Source: Medicine (Baltimore). 2016 Jul 8;95(27):e3825. doi: 10.1097/MD.0000000000003825 (PMC5058789; doi:10.1097/MD.0000000000003825)
Supplement: Supplemental Digital Content [file medi-95-e3825-s001.doc]

**Supplemental Digital Content 1. Retrieval strategy for Chinese and foreign articles**

| **No.** | **Retrieval strategy for Chinese studies** |
| --- | --- |
| **#1** | "induced abortion” [full field] OR "artificial abortion” [full field] OR "abortion, artificial” [unweighted: extension] |
| **#2** | "surgical abortion” [full field] OR "drug abortion” [full field] |
| **#3** | #1 OR #2 |
| **#4** | "oral % contraceptive” [full field] OR “oral % contraceptive pills” [full field] OR “contraceptive, oral” [unweighted: extension] OR (“contraceptive” [unweighted: extension] AND “medication, oral” [unweighted: extension]) |
| **#5** | “drospirenone%ethinyloestradiol” [full field] OR “Yasmin” [full field] OR “YAZ” [full field] OR “Yasmin” [Chinese headline] OR “Yasmin” [key words] OR “Yasmin” [abstract] OR “Yasmin” [full field] OR “oral YAZ” [full field] OR “ethinyloestradiol % drospirenone” [full field] |
| **#6** | “estradiol%drospirenone” [full field] OR “Drospirenone” [English Headline] OR “Drospirenone” [abstract] OR “Safyral” [English Headline] OR “Safyral” [abstract] OR “Beyaz” [English Headline] OR “Beyaz” [abstract] OR “metafolin” [English Headline] OR “metafolin” [abstract] OR “angeliq” [full field] |
| **#7** | norethynodrel [full field] OR "norethynodrel” [unweighted: extension] OR Levonorgestrel [full field] OR Norethisterone [full field] OR "Norethindrone” [full field] OR "Norethisterone” [full field] OR "Norethisterone ” [unweighted: extension] OR Norgestrel [full field] OR levonorgestrel [full field] "L - Norgestrel” [full field] OR "levonorgestrel” [unweighted: extension] OR Yuting levonorgestrel tablets [Chinese headline] OR Yuting levonorgestrel tablets [abstract] OR Anting levonorgestrel tablets [Chinese headline] OR Anting levonorgestrel tablets [Chinese headline] OR Huiting levonorgestrel tablets [Chinese headline] Huiting levonorgestrel tablets [Chinese headline] OR Levonorgestrel quinestrol tablets [full field] OR Baoshiting ostinor levonorgestrel tablets [full field] OR Nuoshuang levonorgestrel (LNG) [full field] OR “Norgestrel” [full field] OR “Ovral” [full field] OR “Planovar” [full field] OR “FecomFe” [full field] OR “Norethisterone” [full field] OR “Micronor” [full field] OR “Jolivette” [full field] OR “Micronor” [full field] OR “Norlutin” [full field] OR “Primolut N” [full field] OR “fumarate” [full field] OR “Femcon Fe” [full field] |
| **#8** | desogestrel ethinyloestradiol [full field] OR Marvelon [full field] OR Marvelon [full field] OR Mercilon [full field] OR “Cyclessa” [full field] OR “Desogen” [full field] OR “Dueva” [full field] OR “Marvelon” [full field] OR “Mercilon” [full field] OR “Microdiol” [full field] OR “Mircette” [full field] OR “Planum” [full field] OR Securgin” [full field] |
| **#9** | levonorgestrel ethinyloestradiol triphasic [full field] OR triquilar [full field] OR Trinordiol [full field] OR Kalirui [full field] OR triphasic contraceptive tablets [full field] OR “Levlite” [full field] OR “Librel” [full field] OR “LoSeasonique” [full field] OR “Lybrel” [full field] OR “Microgynon” [full field] OR “Miranova” [full field] OR “Nordette” [full field] OR “Ovranette” [full field] OR “Quartette” [full field] OR “Seasonale” [full field] OR “Seasonique” [full field] OR “Triphasil” [full field] OR “Triquilar” [full field] OR "Levonorgestrel” [full field] OR “Microval” [full field] |
| **#10** | megestrol acetate [full field] OR "megestrol acetate” [unweighted: extension] OR "AROCK “ [full field] OR "Jiadi megestrol acetate capsules” [Chinese headline] OR "Jiadi megestrol acetate capsules” [abstract] OR "megestrol acetate” [full field] OR megestrol [full field] OR megestrol acetate [full field] OR corporin [full field] OR "Minigest" [full field] OR "Niagestin" [full field] OR "Ovaban” [full field] OR "Ovarid” [full field] OR "Volplan” [full field] OR "Trimegestone” [full field] OR "Ondeva” [full field] |
| **#11** | Gestodene [full field] OR minulet [full field] OR compound gestodene [full field] OR "Convaden" [full field] OR "Femodene” [full field] OR "Ginoden” [full field] OR "Lindynette” [full field] OR "Melodia” [full field] OR "Minesse” [full field] OR "Minulet” [full field] OR "Sunya” [full field] OR "Femovan” [full field] OR "Meliane” [full field] |
| **#12** | "desodestrel” [full field] OR "desogestrel “ [full field] OR "Desogestrel” [full field] OR "desogestrel” [full field] OR "desogestrel” [unweighted: extension] OR "Cerazette” [full field] |
| **#13** | "Diane - 35” [full field] OR "compound cyproterone acetate” [full field] OR "ethinylestradiol and cyproterone acetate” [full field] OR "ethinylestradiol and cyproterone acetate” [full field] OR "norethisterone acetate” [full field] OR "Norethindrone” [full field] OR "Norethisterone” [full field] OR "LOESTRIN” [full field] OR "Microgestin” [full field] OR "Estrostep” [full field] OR "ethinyloestradiol” [full field] OR "ethinyloestradiol” [unweighted: extension] OR "Diane - 35” [full field] OR "Dianette” [full field] |
| **#14** | "norgestimate” [full field] OR "norethisterone acetate oxime” [full field] OR "dienogest” [full field] OR "dienogest” [full field] OR "Norgestimate” [full field] OR "Cilest” [full field] OR "Ortho Tri - Cyclen” [full field] OR "Ortho - Cyclen” [full field] OR "Previfem” [full field] OR "TriNessa” [full field] OR "FecomFe” [full field] OR "Lunabell” [full field] OR "Ovcon” [full field] OR "Zenchent” [full field] OR "Ortho Evra” [full field] |
| **#15** | "Norgestrel “ [full field] OR "chlormadinone” [full field] OR "dienogest” [full field] OR "nomegesttol acetate” [full field] OR "oestradiol valerate” [full field] OR "Zoely” [full field] OR ("Estradiol” [full field] AND valerate [full field]) OR "Klimodien” [full field] OR "Climodien” [full field] OR "Klimodien” [full field] OR "Lafamme” [full field] OR "Natazia” [full field] OR "Qlaira” [full field] |
| **#16** | "OMATE” [full field] OR "WAY - 160910” [full field] OR "BRL - 5” [full field] OR "BRL - 6” [full field] OR "JNJ - 550056” [full field] OR "DR - 1031” [full field] OR "Pill - Plus” [full field] |
| **#17** | #4 OR #5 OR #6 OR #7 OR #8 OR #9 OR #10 OR #11 OR #12 OR #13 OR #14 OR #15 OR #16 |
| **#18** | #3 AND #17 |
| **#19** | #18 AND (translated texts [type of document] OR lecture [type of document] OR summary [type of document]) |
| **#20** | #18 AND (clinical test [type of document] OR randomized controlled trial [type of document] OR Meta analysis [type of document] OR multi-center study [type of document] OR case report [type of document]) |
| **#21** | #18 AND (animal [feature words]) |
| **#22** | #18 AND (human beings [feature words]) |
| **#23** | #1 NOT ((#19 NOT #20) OR (#21 NOT #22)) |

Retrieval strategy of foreign languages

| **No.** | **Retrieval strategy** |
| --- | --- |
| **#1** | Induced Abortion OR "induced abortion” [Mesh] |
| **#2** | Surgery Abortion OR Medical Abortion OR Drug Abortion |
| **#3** | #1 OR #2 |
| **#4** | “oral contraceptive” OR "Contraceptives, Oral” [Mesh] OR ("Contraceptives” [Mesh] AND "Administration, Oral” [Mesh]) |
| **#5** | “Drospirenone Ethinylestradiol” OR Yasmin OR YAZ |
| **#6** | “Drospirenone ethinylestradiol” **[Title / Abstract]** OR Drospirenone [**Title / Abstract]** OR Safyral [**Title / Abstract]** OR Beyaz**[Title / Abstract]** OR metafolin [**Title / Abstract]** |
| **#7** | Norethylnodrel [**Title / Abstract]** OR "norethynodrel” [MeSH] OR "levonorgestrel” [MeSH] OR levonorgestrel [**Title / Abstract]** OR "norethindrone” [MeSH] OR norethindrone [**Title / Abstract]** OR "norgestrel” [MeSH] OR norgestrel [**Title / Abstract]** OR "levonorgestrel” [MeSH] OR levonorgestrel [**Title / Abstract]** OR Ovral [**Title / Abstract]** OR Planovar [**Title / Abstract]** OR FecomFe [**Title / Abstract]** OR IKH - 01**[Title / Abstract]** OR Norethisterone [**Title / Abstract]** OR Micronor [**Title / Abstract]** OR "NX - 200" **[Title / Abstract]** OR "NSC - 9564” [**Title / Abstract]** OR Jolivette [**Title / Abstract]** OR Microno [**Title / Abstract]**r OR Norlutin [**Title / Abstract]** OR “Primolut N” [**Title / Abstract]** OR fumarate [**Title / Abstract]** OR “Femcon Fe” [**Title / Abstract]** |
| **#8** | "Ethinyl estradiol desogestrel” [**Title / Abstract]** OR ("ethinyl estradiol” [MeSH] AND "desogestrel” [MeSH]) OR “Desogestrel ethinylestradiol” **[Title / Abstract]** OR Cyclessa [**Title / Abstract]** OR Desogen [**Title / Abstract]** OR Dueva [**Title / Abstract]** OR Marvelon [**Title / Abstract]** OR Mercilon [**Title / Abstract]** OR Microdiol [**Title / Abstract]** OR Mircette [**Title / Abstract]** OR Planum [**Title / Abstract]** OR Securgin [**Title / Abstract]1611** |
| **#9** | "levonorgestrel” [MeSH Terms] OR levonorgestrel [**Title / Abstract]** OR "AG - 200” [**Title / Abstract]** OR "AG - 200 - 15” [**Title / Abstract]** OR "BRL - 7” [**Title / Abstract]** OR "DP - 3” [**Title / Abstract]** OR "DR - 103” [**Title / Abstract]** OR DR – 105 **[Title / Abstract]** OR Alesse [**Title / Abstract]** OR "Ange28” [**Title / Abstract]** OR Leios [**Title / Abstract]** OR Levlite [**Title / Abstract]** OR “Libian28” **[Title / Abstract]** OR Librel [**Title / Abstract]** OR LoSeasonique [**Title / Abstract]** OR Lybrel [**Title / Abstract]** OR Microgynon [**Title / Abstract]** OR Miranova [**Title / Abstract]** OR Nordette [**Title / Abstract]** OR "Nordette - 28” [**Title / Abstract]** OR Ovranette [**Title / Abstract]** OR Preven [**Title / Abstract]** OR Quartette [**Title / Abstract]** OR Seasonale [**Title / Abstract]** OR Seasonique [**Title / Abstract]** OR "Tridiol 21” [**Title / Abstract]** OR Triphasil [**Title / Abstract]** OR Triquilar [**Title / Abstract]** OR "Triquilar 21” [**Title / Abstract]** OR "Triquilar 28” [**Title / Abstract]** OR Twirla [**Title / Abstract]** OR Microval [**Title / Abstract]** |
| **#10** | "megestrol” [MeSH] OR megestrol [**Title / Abstract]** OR Niagestin [**Title / Abstract]** OR Ovaban [**Title / Abstract]** OR OvariD [**Title / Abstract]** OR Volplan [**Title / Abstract]** OR "trimegestone” [Supplementary Concept] OR trimegestone [**Title / Abstract]** OR Ondeva [**Title / Abstract]** OR "Ru - 27987” [**Title / Abstract]** OR Desogestrel [**Title / Abstract]** OR Cerazette [**Title / Abstract]** OR "Org - 296” [**Title / Abstract]** |
| **#11** | "Gestodene” [Supplementary Concept] OR Gestodene [**Title / Abstract]** OR "BAY - 86 - 5016” [**Title / Abstract]** OR "SH - 543” [**Title / Abstract]** OR Arianna [**Title / Abstract]** OR Convaden [**Title / Abstract]** OR Fedra [**Title / Abstract]** OR Femodene [**Title / Abstract]** OR Ginoden [**Title / Abstract]** OR Katya [**Title / Abstract]** OR Lindynette [**Title / Abstract]** OR Melodia [**Title / Abstract]** OR Minesse [**Title / Abstract]** OR Minulet **[Title / Abstract]** OR Sunya **[Title / Abstract]** OR Femovan **[Title / Abstract]** OR Meliane **[Title / Abstract]** |
| **#12** | "desogestrel” [MeSH Terms] OR desogestrel [**Title / Abstract]** OR Cerazette [**Title / Abstract]** |
| **#13** | "norethindrone” [MeSH Terms] OR norethindrone **[Title / Abstract]** OR Norethisterone **[Title / Abstract]** OR "LOESTRIN FE" **[Title / Abstract]** OR "microcystin” [Supplementary Concept] OR microcystin **[Title / Abstract]** OR "Estrostep 21” [**Title / Abstract]** OR "WC - 3016” [**Title / Abstract]** OR "Estrostep Fe" **[Title / Abstract]** OR "Lo Loestrin Fe” [**Title / Abstract]** OR "Lo Minastrin Fe” [**Title / Abstract]** OR "Loestrin 24 Fe" **[Title / Abstract]** OR "Loestrin Fe 1.5 / 30” [**Title / Abstract]** OR "Loestrin Fe 1 / 20” [**Title / Abstract]** OR "Minastrin 24 Fe” [**Title / Abstract]** OR "Ortho 777 - 21” [**Title / Abstract]** OR "Ortho M - 21” [**Title / Abstract]** OR "Synphase T28" **[Title / Abstract]** OR "Diane - 35” [**Title / Abstract]** OR "cyproterone acetate” [MeSH Terms] OR "cyproterone acetate" OR "Estrostep Fe” [**Title / Abstract]** OR "Lo Loestrin Fe” [**Title / Abstract]** OR "Loestrin 24 Fe” [**Title / Abstract]** OR "MINASTRIN FE” [**Title / Abstract]** OR Valette [**Title / Abstract]** OR Dianette [**Title / Abstract]** |
| **#14** | "norgestimate” [Supplementary Concept] OR "norgestimate" OR Cilest**[Title / Abstract]** OR "Ortho Tri - Cyclen” [**Title / Abstract]** OR "Ortho Tri - Cyclen LO” [**Title / Abstract]** OR "Ortho - Cyclen” [**Title / Abstract]** OR Previfem [**Title / Abstract]** OR "Tri - Previfem” [**Title / Abstract]** OR TriNessa [**Title / Abstract]** OR "IKH - 01” [**Title / Abstract]** OR "NPC - 01” [**Title / Abstract]** OR "FecomFe” [**Title / Abstract]** OR Lunabell [**Title / Abstract]** OR "Necon 7 / 7 / 7” [**Title / Abstract]** OR "Ortho - Novum 1 / 35” [**Title / Abstract]** OR "Ortho - Novum 10 / 11” [**Title / Abstract]** OR "Ortho - Novum 7 / 7 / 7” [**Title / Abstract]** OR Ovcon [**Title / Abstract]** OR Zenchent**[Title / Abstract]** |
| **#15** | "norgestrel” [MeSH Terms] OR "norgestrel” [**Title / Abstract]** OR "chlormadinone acetate” [MeSH Terms] OR "chlormadinone acetate” [**Title / Abstract]** OR "dienogest” [Supplementary Concept] OR dienogest**[Title / Abstract]** OR "estradiol valerate” [Supplementary Concept] OR "estradiol valerate” [**Title / Abstract]** OR "E2 / Nomac” [**Title / Abstract]** OR "EMM - 210066” [**Title / Abstract]** OR "EMM - 220066” [**Title / Abstract]** OR "EMM - 310066” [**Title / Abstract]** OR "MK - 8175A” [**Title / Abstract]** OR "Nomac / E2” [**Title / Abstract]** OR "SCH - 900121” [**Title / Abstract]** OR "TX - 45066” [**Title / Abstract]** OR Naemis **[Title / Abstract]** OR Zoely**[Title / Abstract]** OR Klimodien **[Title / Abstract]** OR "BAY - 86 - 5027” [**Title / Abstract]** OR "SH - T00658ID” [**Title / Abstract]** OR Climodien **[Title / Abstract]** OR Klimodien **[Title / Abstract]** OR Lafamme **[Title / Abstract]** OR Natazia **[Title / Abstract]** OR Qlaira **[Title / Abstract** |
| **#16** | OMATE [**Title / Abstract]** OR "WAY - 160910" **[Title / Abstract]** OR "BRL - 5” [**Title / Abstract]** OR "BRL - 6” [**Title / Abstract]** OR "JNJ - 550056” [**Title / Abstract]** OR "DR - 1031” [**Title / Abstract]** OR "Pill - Plus" |
| **#17** | #4 OR #5 OR #6 OR #7 OR #8 OR #9 OR #10 OR #11 OR #12 OR #13 OR #14 OR #15 OR #16 |
| **#18** | #3 AND #17 |
| **#19** | #18 AND Filters: Comment; Editorial; Review; News |
| **#20** | #18 AND Filters: Case Reports; Clinical Trial; Comparative Study; Controlled Clinical Trial; Observational Study; Multicenter Study; Evaluation Studies; Randomized Controlled Trial |
| **#21** | #18 AND Filters: Other Animals |
| **#22** | #18 AND Filters: Humans |
| **#23** | #18 NOT ((#19 NOT #20) OR (#21 NOT #22)) |

**Supplemental Digital Content 2. Relative Indicator Data of Inclusion Research – 1^23-141^**

| **Included research** | **Sample  Size** | **Abortion and Medicine Taking Plan** | **Colporrhagia  time (d)** | | **Colporrhagia  amount(ml)** | | **The period till  the next  menstruation (d)** | | **Endometrial  thickness 2 weeks after abortion** | | **Endometrial  thickness 3 weeks after abortion** | |
| --- | --- | --- | --- | --- | --- | --- | --- | --- | --- | --- | --- | --- |
|  |  |  | **Research  group** | **Control  group** | **Research  group** | **Control  group** | **Research  group** | **Control group** | **Research group** | **Control group** | **Research group** | **Control group** |
| Liu Zheng-ping,  2000 | 101  (59 / 42) | ①③ | 13.60 ± 6.48 | 16.70 ± 8.21 | 40.08 ± 23.17 | 50.97 ± 26.70 |  |  |  |  |  |  |
| Liu Zheng-ping, 2004 | 183  (93 / 90) | ①③ | 12.40 ± 6.89 | 16.10 ± 8.12 | 41.21 ± 24.22 | 52.31 ± 27.42 |  |  |  |  |  |  |
| Li Yan-fang, 1998 | 183 (68 / 115) | ①③ | 17.47 ± 6.49 | 14.79 ± 6.36 |  |  |  |  |  |  |  |  |
| Gong Mei-xuan, 2008 | 134  (66 / 68) | ①③ | 9.2 ± 1.5 | 13.7 ± 2.6 | 60.8 ± 1.7 | 91.7 ± 5.3 |  |  |  |  |  |  |
| Lu Yun, 2009 | 80  (40 / 40) | ①③ | 10.32 ± 5.33 | 15.15 ± 6.73 |  |  |  |  |  |  |  |  |
| Wang Cai-yan, 2011 | 135  (41 / 94) | ①③ | 20 ± 13 | 19 ± 11 |  |  | 34 ± 13 | 39 ± 11 |  |  |  |  |
| Deng Xue-bin, 2013 | 76  (38 / 38) | ①③ | 8.33 ± 2.16 | 11.94 ± 2.41 |  |  | 27.05 ± 2.49 | 35.29 ± 3.48 |  |  |  |  |
| Yang Qin-ling, 2010 | 206  (103 / 103) | ①③ | 10.3 ± 5.7 | 16.8 ± 7.2 |  |  |  |  |  |  |  |  |
| Lu Xiu-fang, 2007 | 200  (100 / 100) | ①③ | 10.54 ± 6.32 | 16.63 ± 7.54 |  |  |  |  |  |  |  |  |
| Chen Qin-fang,  2011 | 414  (209 / 205) | ①③ | 17.5 ± 7.4 | 18.1 ± 9.6 |  |  |  |  |  |  |  |  |
| Zhang Ling-juan,  2012 | 216 (114 / 72) | ①③ |  |  |  |  | 25.9 ± 1.1 | 36.9 ± 1.74 |  |  |  |  |
| Huang Qiu-lian,  2009 | 200 (100 / 100) | ①③ | 5.50 ± 2.45 | 7.25 ± 2.85 |  |  |  |  |  |  |  |  |
| Pu Wen-ying, 2009 | 14775 / 72 () | ①③ |  |  |  |  |  |  |  |  |  |  |
| Zhu Jing, 2014 | 150 (75 / 75) | ①③ | 3.5 ± 1.6 | 6.7 ± 2.2 |  |  | 30.5 ± 4.6 | 39.6 ± 5.1 |  |  |  |  |
| Jin Rui-ying, 2013 | 120 (60 / 60) | ①③ | 5.4 ± 1.5 | 8.1 ± 1.7 | 40.3 ± 7.2 | 58.7 ± 10.2 | 28.1 ± 2.7 | 34.2 ± 3.1 |  |  | 8.7 ± 1.6 | 6.4 ± 1.1 |
| Sun Ke-li, 2012 | 100 (50 / 50) | ①③ |  |  |  |  |  |  | 7.2 ± 2.6 | 4.9 ± 1.7 |  |  |
| Zhang Jin, 2011 | 120 (60 / 60) | ①③ | 5.23 ± 2.35 | 6.89 ± 3.2 |  |  | 25.50 ± 3.11 | 37.40 ± 7.15 |  |  |  |  |
| Chen Yu,  2010 | 200 (100 / 100) | ①③ | 5.50 ± 2.45 | 7.25 ± 2.85 |  |  |  |  |  |  |  |  |
| Guo Fang-hua, 2014 | 220 (110 / 110) | ①③ | 5.0 ± 1.5 | 7.2 ± 1.6 |  |  | 29.42 ± 3.24 | 34.62 ± 2.91 |  |  |  |  |
| Bai Feng, 2014 | 100 (50 / 50) | ①③ | 3.67 ± 2.56 | 5.38 ± 3.18 |  |  | 30.55 ± 3.24 | 36.47 ± 3.58 |  |  |  |  |
| Xu Wan-li, 2012 | 360 (180 / 180) | ①③ |  |  |  |  |  |  |  |  |  |  |
| Dong Li-xiang,  2014 | 106 (53 / 53) | ①③ | 5.2 ± 1.3 | 7.1 ± 2.2 |  |  |  |  |  |  |  |  |
| Yu Jian-li, 2014 | 280 (140 / 140) | ①③ | 4.72 ± 1.23 | 6.90 ± 1.55 | 41.18 ± 7.59 | 55.76 ± 9.24 | 28.67 ± 6.25 | 34.42 ± 7.06 |  |  |  |  |
| Tan Jing,  2014 | 150 (75 / 75) | ①③ | 4.61 ± 1.13 | 6.89 ± 1.44 | 40.23 ± 6.89 | 58.46 ± 9.14 |  |  |  |  |  |  |
| Zhuang Lin, 2008 | 166 (84 / 82) | ①③ |  |  |  |  |  |  |  |  |  |  |
| Xue Xia,  2012 | 900 (600 / 300) | ①③ | 3.85 ± 2.45 | 6.3 ± 3.4 | 24.7 ± 3.12 | 25.1 ± 2.8 | 23 ± 3.62 | 32.1 ± 4.1 | 5.25 ± 2.2 | 4.9 ± 2.5 |  |  |
| Wu Chen-dan, 2012 | 120 (60 / 60) | ①③ |  |  |  |  |  |  |  |  |  |  |
| Gu Xiao-yan, 2012 | 100 (50 / 50) | ①③ | 8 ± 2.5 | 14 ± 3.4 |  |  |  |  |  |  |  |  |
| Wang Min, 2012 | 136 (68 / 68) | ①③ |  |  |  |  | 28.5 ± 2.1 | 37.7 ± 4.3 |  |  |  |  |
| Zhu Wei-na, 2014 | 220 (110 / 110) | ①③ | 4.8 ± 3.1 | 7.4 ± 5.2 |  |  | 26.7 ± 5.6 | 37.5 ± 3.2 |  |  |  |  |
| Mao Dan-qing,  2013 | 140 (71 / 69) | ①③ | 5.7 ± 1.3 | 8.6 ± 1.8 |  |  | 28.1 ± 3.7 | 35.7 ± 4.5 |  |  | 8.9 ± 1.8 | 6.5 ± 1.2 |
| Xia Hong-xia, 2014 | 408 (204 / 204) | ①③ |  |  |  |  |  |  |  |  | 6.2 ± 2.0 | 5.0 ± 1.8 |
| Sun Dong-yan, 2014 | 400 (200 / 200) | ①③ |  |  |  |  |  |  |  |  |  |  |
| Ding Xiao-hong,  2011 | 100 (50 / 50) | ①③ |  |  |  |  | 28 ± 2.13 | 37 ± 5.23 |  |  |  |  |
| Rui Yu-xuan, 2011 | 1140 (530 / 610) | ①③ | 4.36 ± 1.25 | 6.64 ± 3.23 |  |  | 25.65 ± 2.87 | 36.35 ± 7.24 | 5.31 ± 1.53 | 4.73 ± 1.89 |  |  |
| Li Cui-fen, 2011 | 200 (100 / 100) | ①③ |  |  |  |  |  |  |  |  | 9.08 ± 0.29 | 6.95 ± 0.98 |
| Chen Yan, 2014 | 460 (230 / 230) | ①③ | 8 ± 4 | 12 ± 5 | 35 ± 5 | 52 ± 4 | 29 ± 3 | 38 ± 7 |  |  |  |  |
| Li Hui-rong, 2013 | 1189 (603 / 586) | ①③ | 6.3 ± 1.4 | 7.0 ± 2.7 |  |  | 27.1 ± 1.5 | 32.3 ± 6.5 |  |  | 7.4 ± 2.5 | 5.4 ± 2.7 |
| Ding Hai-qian, 2014 | 200 (100 / 100) | ①③ | 3.6 ± 2.4 | 6.5 ± 3.3 | 24.2 ± 3.0 | 25.2 ± 2.7 | 23.6 ± 3.3 | 33.8 ± 4.1 | 5.3 ± 2.1 | 4.9 ± 2.4 |  |  |
| Yang Jin-qing, 2011 | 240 (120 / 120) | ①③ |  |  |  |  |  |  |  |  |  |  |
| Xue Xia,  2012 | 600 (300 / 300) | ①③ | 3.8 ± 2.4 | 6.3 ± 3.4 | 24.3 ± 3.1 | 25.1 ± 2.8 | 23.2 ± 3.2 | 32.1 ± 4.1 | 5.2 ± 2.1 | 4.9 ± 2.5 |  |  |
| Guo Yi-hong, 2010 | 200 (100 / 100) | ②④ |  |  |  |  | 30 ± 4 | 39 ± 5 |  |  |  |  |
| Zhuang Shui-lian,  2012 | 400 (200 / 200) | ②④ |  |  |  |  |  |  |  |  |  |  |
| Guo Yin-e, 2012 | 160 (80 / 80) | ②④ |  |  |  |  |  |  |  |  |  |  |
| Zhang Lin-lin, 2014 | 305 (150 / 105) | ②④ |  |  |  |  |  |  |  |  |  |  |
| Wen Hua-ying,  2014 | 120 (60 / 60) | ②④ |  |  |  |  |  |  |  |  |  |  |
| Liu Jian-wen, 2010 | 119 (60 / 59) | ②④ | 5.2 ± 1.2 | 7.9 ± 2.5 |  |  | 26.2 ± 1.2 | 31.5 ± 3.6 |  |  |  |  |
| Sun Yang, 2014 | 180 (90 / 90) | ②④ |  |  |  |  |  |  |  |  |  |  |
| Pan Cui-hong, 2013 | 162 (81 / 81) | ②④ | 5.48 ± 1.65 | 7.16 ± 1.83 |  |  | 27.35 ± 2.58 | 30.42 ± 3.42 |  |  |  |  |
| Xu Hong, 2014 | 260 (180 / 180) | ②④ | 4.31 ± 1.80 | 6.60 ± 2.42 |  |  | 26.12 ± 3.80 | 36.92 ± 6.81 |  |  |  |  |
| Zhan An-na, 2014 | 100 (50 / 50) | ②④ | 4.56 ± 1.10 | 6.81 ± 1.16 | 43.23 ± 12.10 | 54.34 ± 13.22 | 27.31 ± 3.51 | 33.26 ± 3.02 |  |  |  |  |
| Huang Li-guang,  2014 | 240 (120 / 120) | ②④ |  |  |  |  |  |  |  |  |  |  |
| Zong Ji-wei, 2013 | 100 (50 / 50) | ②④ | 5.70 ± 1.33 | 8.00 ± 1.17 |  |  | 28.55 ± 3.01 | 37.87 ± 3.64 |  |  |  |  |
| Han Ying, 2013 | 180 (90 / 90) | ②④ | 4.3 ± 1.2 | 6.5 ± 2.9 |  |  | 26.7 ± 4.2 | 29.8 ± 5.6 |  |  |  |  |
| Luo Ning, 2013 | 180 (90 / 90) | ②④ |  |  |  |  |  |  |  |  |  |  |
| Zhang Dong-ling, 2013 | 145 (70 / 70) | ②④ |  |  |  |  |  |  | 7.3 ± 2.0 | 4.7 ± 1.8 |  |  |
| Wang Ju-hui 2013 | 300 (150 / 150) | ②④ |  |  |  |  |  |  |  |  |  |  |
| Yao Wen-lei, 2013 | 152 (76 / 76) | ②④ | 4.54 ± 1.18 | 8.32 ± 2.75 |  |  |  |  |  |  |  |  |
| Lu Ling,  2013 | 210 (105 / 105) | ②④ |  |  |  |  |  |  |  |  |  |  |
| Chen Yan-hong, 2013 | 178 (89 / 89) | ②④ | 5.1 ± 1.3 | 7.8 ± 2.1 |  |  | 25.9 ± 1.5 | 32.1 ± 2.9 | 6.3 ± 1.9 | 4.2 ± 1.6 |  |  |
| Li Ya-jun, 2012 | 120 (60 / 60) | ②④ | 3.35 ± 0.67 | 4.87 ± 0.48 |  |  | 28.55 ± 3.01 | 37.87 ± 3.64 |  |  |  |  |
| Shen Xiu-xiang, 2012 | 120 (60 / 60) | ②④ | 2.9 ± 0.6 | 3.8 ± 1.0 |  |  | 28.0 ± 2.1 | 37.0 ± 5.3 |  |  |  |  |
| Xiang Dong-shun, 2012 | 200 (100 / 100) | ②④ | 5.2 ± 1.3 | 7.0 ± 1.7 |  |  |  |  |  |  |  |  |
| Zhang Zhong-hua, 2012 | 224 (112 / 112) | ②④ |  |  |  |  |  |  |  |  |  |  |
| Chen Li,  2012 | 400 (200 / 200) | ②④ | 5.1 ± 1.9 | 9.3 ± 2.35 |  |  |  |  |  |  | 6.7 ± 2.1 | 5.1 ± 1.9 |
| Xu Yan-li, 2012 | 320 (160 / 160) | ②④ |  |  |  |  |  |  |  |  |  |  |
| Zou Xia, 2012 | 200 (100 / 100) | ②④ |  |  |  |  |  |  |  |  |  |  |
| Fan Jin-chan, 2012 | 140 (70 / 70) | ②④ | 5.25 ± 2.25 | 7.45 ± 2.55 |  |  |  |  |  |  |  |  |
| Xu Yun-e, 2012 | 240 (120 / 120) | ②④ |  |  |  |  |  |  |  |  |  |  |
| Yao Ling, 2011 | 210 (105 / 105) | ②④ |  |  |  |  | 27.9 ± 4.2 | 36.1 ± 6.9 |  |  |  |  |
| Hou Lian-yun, 2011 | 160 (80 / 80) | ②④ | 5.43 ± 2.35 | 7.23 ± 3.01 |  |  | 26.30 ± 3.4 | 34.40 ± 6.4 |  |  |  |  |
| Lv Xiao-lan, 2011 | 100 (50 / 50) | ②④ |  |  |  |  |  |  |  |  |  |  |
| Wu Cheng-zhen,  2011 | 400 (200 / 200) | ②④ |  |  |  |  | 30.2 ± 4.1 | 35.6 ± 6.4 |  |  |  |  |
| Li Xin-ting, 2011 | 170 (85 / 85) | ②④ | 4.32 ± 1.17 | 7.38 ± 2.86 |  |  | 34.67 ± 8.85 | 42.98 ± 9.45 |  |  |  |  |
| Wang Wen-rong, 2011 | 240 (120 / 120) | ②④ |  |  |  |  |  |  |  |  |  |  |
| Meng Xiao-yan, 2011 | 666 (320 / 346) | ②④ |  |  |  |  |  |  |  |  |  |  |
| Li Jian-xiu, 2010 | 128 (64 / 64) | ②④ | 6.4 ± 1.7 | 7.2 ± 1.2 |  |  | 31.0 ± 2.2 | 35.2 ± 6.4 |  |  |  |  |
| Hu Ting, 2009 | 260 (130 / 130) | ②④ |  |  |  |  |  |  |  |  |  |  |
| Du Ai-qi,2009 | 550 (274 / 276) | ②④ | 7.5 ± 1.5 | 8.2 ± 1.5 |  |  | 30.5 ± 1.5 | 36.5 ± 4.5 |  |  |  |  |
| Zhao Cai-qin, 2011 | 2209110 / 110) | ②④ |  |  |  |  | 28.5 ± 4.8 | 29.2 ± 5.2 |  |  |  |  |
| Qin Li-na, 2012 | 300 (150 / 150) | ②④ | 7 ± 6 | 12 ± 3 | 43 ± 12 | 55 ± 12 |  |  |  |  |  |  |
| Guo Yu-sheng, 2013 | 100 (50 / 50) | ②④ | 6.5 ± 1.8 | 7.6 ± 1.4 |  |  | 30.9 ± 2.8 | 36.1 ± 4.3 |  |  |  |  |
| Wang Xu-yin, 2011 | 385 (185 / 200) | ②④ | 1.01 ± 0.84 | 2.59 ± 0.96 |  |  | 27.32 ± 2.17 | 35.91 ± 2.83 |  |  |  |  |
| Tang Yong-zhen, 2014 | 110 (55 / 55) | ②④ | 5.0 ± 1.2 | 7.7 ± 1.9 |  |  | 24.3 ± 1.7 | 32.5 ± 2.5 | 6.4 ± 1.8 | 4.1 ± 1.5 |  |  |
| Zeng Yin-ying, 2013 | 100 (50 / 50) | ②④ | 3.90 ± 2.1 | 5.54 ± 3.02 |  |  | 30.8 ± 3.53 | 37.52 ± 6.33 |  |  |  |  |
| Ou Ji-lan, 2014 | 200 (100 / 100) | ②④ | 3.26 ± 1.40 | 5.27 ± 2.31 |  |  | 31.86 ± 10.62 | 35.97 ± 10.43 |  |  |  |  |
| Yang Yong, 2014 | 184 (94 / 90) | ②④ | 6.2 ± 1.0 | 7.8 ± 1.4 |  |  | 29.8 ± 2.5 | 34.2 ± 3.1 |  |  |  |  |
| Yu Chan-yang, 2014 | 160 (80 / 80) | ②④ |  |  |  |  |  |  |  |  |  |  |
| Zhang Yuan, 2014 | 240 (120 / 120) | ②④ | 3.8 ± 1.2 | 7.4 ± 1.8 |  |  | 28.7 ± 4.8 | 37.2 ± 6.1 |  |  |  |  |
| Lei Xun, 2012 | 180 (90 / 90) | ②④ |  |  |  |  | 28.9 ± 4.4 | 35.4 ± 6.1 |  |  |  |  |
| Han Dong-hong, 2012 | 300 (150 / 150) | ②④ | 5.78 ± 0.48 | 6.86 ± 0.57 | 47.82 ± 2.77 | 82.27 ± 2.86 | 24.00 ± 2.24 | 36.81 ± 7.79 |  |  |  |  |
| Xie Feng, 2009 | 379 (198 / 199) | ②④ | 6.3 ± 3.4 | 5.9 ± 2.3 |  |  | 23.3 ± 3.1 | 30.0 ± 4.6 | 5.2 ± 2.6 | 4.9 ± 1.7 |  |  |
| Chen Si-feng,2013 | 240 (120 / 120) | ②④ |  |  |  |  |  |  |  |  |  |  |
| Zhang  Chang-qing,  2011 | 274 (149 / 98) | ②④ | 3.79 ± 0.72 | 7.2 ± 1.1 |  |  | 25.89 ± 1.1 | 30.1 ± 2.9 |  |  |  |  |
| Zhang Chun-yan, 2013 | 480 (240 / 240) | ②④ |  |  |  |  | 27 ± 4 | 42 ± 5 |  |  |  |  |
| Rui Hong-wei, 2013 | 632 (318 / 314) | ②④ |  |  |  |  | 27.3 ± 3.1 | 36.1 ± 5.9 |  |  | 8.5 ± 0.4 | 6.1 ± 0.3 |
| Zhang Ying-hui, 2013 | 105 (50 / 55) | ②④ | 6.86 ± 5.32 | 7.03 ± 4.74 |  |  | 25 ± 3.21 | 38 ± 4.46 |  |  |  |  |
| Yan Wei-wei, 2013 | 320 (160 / 160) | ②④ |  |  |  |  |  |  |  |  |  |  |
| Feng Ming-yue, 2012 | 160 (80 / 80) | ②④ |  |  |  |  | 31.56 ± 1.88 | 42.79 ± 7.97 |  |  | 10.71 ± 1.80 | 7.98 ± 1.48 |
| Sun Guang-fan,  2013 | 1000 (500 / 500) | ②④ | 6.3 ± 1.8 | 9.0 ± 2.3 |  |  | 27.1 ± 1.2 | 36.4 ± 2.9 | 9.0 ± 1.4 | 6.0 ± 1.2 |  |  |
| Liu Jian-hua, 2014 | 208 (100 / 108) | ②④ | 5.02 ± 1.77 | 7.78 ± 2.34 |  |  | 25.4 ± 2.87 | 37.1 ± 5.07 | 7.32 ± 1.47 | 6.17 ± 1.34 | 10.02 ± 2.39 | 8.07 ± 1.65 |
| Huang Yin, 2014 | 136 (68 / 68) | ②④ |  |  |  |  |  |  | 5.5 ± 2.1 | 4.5 ± 2.1 |  |  |
| Wang Xue-yan,  2014 | 260 (130 / 130) | ②④ |  |  |  |  |  |  |  |  |  |  |
| Lan Chong, 2013 | 200 (100 / 100) | ②④ |  |  |  |  |  |  |  |  |  |  |
| Xia Li-hua, 2013 | 1186 (593 / 593) | ②④ | 5.78 ± 0.48 | 6.86 ± 0.57 | 47.8 ± 2.77 | 82.2 ± 2.86 |  |  |  |  |  |  |
| Ai Yin-hui, 2012 | 140 (70 / 70) | ②④ | 6.1 ± 1.6 | 8.7 ± 1.8 |  |  | 27.8 ± 2.4 | 35.0 ± 6.1 | 7.6 ± 1.2 | 6.0 ± 1.1 |  |  |
| Xue Lei, 2011 | 240 (120 / 120) | ②④ | 5.23 ± 1.89 | 6.68 ± 2.78 |  |  | 27.95 ± 4.11 | 31.11 ± 4.22 |  |  |  |  |
| Meng Xia, 2011 | 120 (60 / 60) | ②④ | 6.9 ± 1.5 | 7.2 ± 1.2 |  |  | 27 ± 1.1 | 36. ± 3.8 | 6.2 ± 2.0 | 5.0 ± 1.8 |  |  |
| Yi Jing-qiu, 2014 | 200 (100 / 100) | ②④ | 6.89 ± 2.52 | 12.23 ± 3.86 |  |  | 28.78 ± 3.56 | 35.56 ± 6.87 |  |  |  |  |
| Zheng Chun-qin, 2014 | 198 (99 / 99) | ②④ | 5.94 ± 0.45 | 7.88 ± 0.62 |  |  | 28.7 ± 3.5 | 34.2 ± 5.7 |  |  |  |  |
| Liu Min, 2014 | 111 (50 / 61) | ②④ | 10.51 ± 2.44 | 12.31 ± 2.64 | 50.21 ± 14.11 | 61.77 ± 15.22 |  |  |  |  |  |  |
| Zhang Li-rong, 2014 | 104  (52 / 52) | ②④ | 8.25 ± 1.57 | 10.13 ± 3.12 | 37.58 ± 10.83 | 53.75 ± 12.17 | 26.69 ± 1.34 | 32.12 ± 4.21 |  |  |  |  |
| Zhang Xin, 2014 | 420  (210 / 210) | ②④ |  |  |  |  | 24 ± 5.1 | 40 ± 6.7 |  |  |  |  |
| Yang Ni, 2013 | 176  (88 / 88) | ②④ | 6 ± 1.2 | 12 ± 2.8 |  |  |  |  |  |  |  |  |
| Zhu Dan-yang, 2014. | 167  (85 / 82) | ②④ | 4 ± 0.6 | 7 ± 1.5 |  |  | 28 ± 3.4 | 35 ± 2.3 |  |  |  |  |
| Zhang Yu-rong, 2014 | 240  (120 / 120) | ②④ | 5.2 ± 1.9 | 6.7 ± 2.8 |  |  |  |  |  |  |  |  |
| Wu Dan-mei,2010 | 200 (100 / 100) | ②④ |  |  |  |  | 28 ± 2.5 | 38.3 ± 3.7 |  |  |  |  |
| Lv Mei, 2013 | 200 (100 / 100) | ②④ |  |  |  |  |  |  |  |  |  |  |
| Zhang Yong-cun, 2013 | 502 (267 / 235) | ②④ | 5.0 ± 2.73 | 12.0 ± 3.64 |  |  |  |  | 9.0 ± 1.62 | 5.2 ± 1.73 |  |  |
| Zhang Fa-li, 2013 | 200 (100 / 100) | ①③ | 5.6 ± 1.4 | 7.5 ± 1.2 |  |  | 26.5 ± 3.8 | 35.1 ± 3.5 |  |  |  |  |

Medicine Taking Plan

①The patients in the research group take OC.

②The patients in the research group take OC and traditional Chinese medicine.

③The patients in the control group take placebo, blank control, condoms, and the couple follow other contraceptive measures.

④The patients in the control group orally take traditional Chinese medicine.

**Supplemental Digital Content 3. Relevant Indicator Data of Included Research - 2^23-141^**

| **Included Research** | **Sample Size** | **Medicine Taking Plan** | **Complication** | | | | **Epicyesis** | |
| --- | --- | --- | --- | --- | --- | --- | --- | --- |
|  |  |  | **Research group (cases)** | **Types of Diseases** | **Control group (cases)** | **Types of Diseases** | **Research group** | **Control group** |
| Liu Zheng-ping, 2000 | 101 (59 / 42) | ①③ |  |  |  |  |  |  |
| Liu Zheng-ping,2004 | 183 (93 / 90) | ①③ |  |  |  |  |  |  |
| Li Yan-fang,1998 | 183 (68 / 115) | ①③ |  |  |  |  |  |  |
| Gong Mei-xuan,2008 | 134 (66 / 68) | ①③ |  |  |  |  |  |  |
| Lu Yun,2009 | 80 (40 / 40) | ①③ |  |  |  |  |  |  |
| Wang Cai-yan,2011 | 135 (41 / 94) | ①③ |  |  |  |  |  |  |
| Deng Xue-bin,2013 | 76 (38 / 38) | ①③ |  |  |  |  |  |  |
| Yang Qin-ling,2010 | 206 (103 / 103) | ①③ |  |  |  |  |  |  |
| Lu Xiu-fang,2007 | 200 (100 / 100) | ①③ |  |  |  |  | 1 | 21 |
| Chen Qin-fang, 2011 | 414 (209 / 205) | ①③ |  |  |  |  |  |  |
| Zhang Ling-juan, 2012 | 216 (114 / 72) | ①③ | 20 | 20 cases of menostasis,  0 case of uterine cavity adhesion | 13 | 10 cases of menostasis, 3 cases of uterine cavity adhesion | 0 | 2 |
| Huang Qiu-lian, 2009 | 200 (100 / 100) | ①③ |  |  |  |  | 0 | 3 |
| Pu Wen-ying, 2009 | 14775 / 72 () | ①③ |  |  |  |  | 1 | 20 |
| Zhu Jing,2014 | 150 (75 / 75) | ①③ |  |  |  |  |  |  |
| Jin Rui-ying, 2013 | 120 (60 / 60) | ①③ | 1 | 1 case of pelvic cavity  infection | 2 | 2 cases of pelvic cavity infection, |  |  |
| Sun Ke-li, 2012 | 100 (50 / 50) | ①③ |  |  |  |  |  |  |
| Zhang Jin, 2011 | 120 (60 / 60) | ①③ |  |  |  |  | 0 | 2 |
| Chen Yu, 2010 | 200 (100 / 100) | ①③ |  |  |  |  | 0 | 3 |
| Guo Fang-hua, 2014 | 220 (110 / 110) | ①③ |  |  |  |  |  |  |
| Bai Feng, 2014 | 100 (50 / 50) | ①③ |  |  |  |  | 0 | 3 |
| Xu Wan-li, 2012 | 360 (180 / 180) | ①③ | 0 | 0 case of cervical adhesion,  0 case of pelvic cavity infection,  0 case of menostasis | 3 | 1 case of cervical adhesion, 1 case of pelvic cavity infection,  1 case of menostasis |  |  |
| Dong Li-xiang, 2014 | 106 (53 / 53) | ①③ |  |  |  |  | 0 | 3 |
| Yu Jian-li, 2014 | 280 (140 / 140) | ①③ |  |  |  |  |  |  |
| Tan Jing, 2014 | 150 (75 / 75) | ①③ |  |  |  |  |  |  |
| Zhuang Lin, 2008 | 166 (84 / 82) | ①③ | 0 | 0 case of uterine cavity adhesion,  0 case of menostasis | 34 | 23 cases of uterine cavity adhesion,  11 case of menostasis | 0 | 3 |
| Xue Xia, 2012 | 900 (600 / 300) | ①③ | 2 | 2 cases of pelvic cavity infection, menostasis 0 case,  0 case of uterine cavity adhesion | 10 | 3 cases of menostasis,  2 cases of uterine cavity adhesion,  5 cases of pelvic cavity infection |  |  |
| Wu Chen-dan, 2012 | 120 (60 / 60) | ①③ | 0 | 0 case of pelvic inflammation | 1 | 1 case of pelvic inflammation | 1 | 9 |
| Gu Xiao-yan, 2012 | 100 (50 / 50) | ①③ | 0 | 0 case of pelvic inflammation | 1 | 1 case of pelvic inflammation |  |  |
| Wang Min, 2012 | 136 (68 / 68) | ①③ |  |  |  |  | 0 | 1 |
| Zhu Wei-na, 2014 | 220 (110 / 110) | ①③ | 2 | 2 cases of uterine cavity adhesion | 10 | 10 cases of uterine cavity adhesion | 0 | 9 |
| Mao Dan-qing, 2013 | 140 (71 / 69) | ①③ |  |  |  |  |  |  |
| Xia Hong-xia, 2014 | 408 (204 / 204) | ①③ |  |  |  |  |  |  |
| Sun Dong-yan, 2014 | 400 (200 / 200) | ①③ | 5 | 5 cases of uterine cavity adhesion | 9 | 9 cases of uterine cavity adhesion |  |  |
| Ding Xiao-hong, 2011 | 100 (50 / 50) | ①③ |  |  |  |  | 0 | 2 |
| Rui Yu-xuan, 2011 | 1140 (530 / 610) | ①③ | 3 | 1 case of menostasis, 2 cases of uterine cavity adhesion | 25 | 6 cases of menostasis,  19 cases of uterine cavity adhesion |  |  |
| Li Cui-fen, 2011 | 200 (100 / 100) | ①③ |  |  |  |  |  |  |
| Chen Yan, 2014 | 460 (230 / 230) | ①③ | 5 | 5 cases of uterine cavity adhesion | 20 | 20 cases of uterine cavity adhesion |  |  |
| Li Hui-rong, 2013 | 1189 (603 / 586) | ①③ | 7 | 7 cases of pelvic inflammation | 46 | 46 cases of pelvic inflammation | 4 | 27 |
| Ding Hai-qian, 2014 | 200 (100 / 100) | ①③ | 1 | 1 case of pelvic inflammation, 0 case of menostasis | 6 | 2 cases of menostasis,  4 cases of pelvic inflammation | 0 | 1 |
| Yang Jin-qing, 2011 | 240 (120 / 120) | ①③ |  |  |  |  | 1 | 37 |
| Xue Xia, 2012 | 600 (300 / 300) | ①③ | 1 | 1 case of pelvic cavity infection, 0 case of menostasis,  uterine cavity adhesion 0 case | 10 | 3 cases of menostasis,  2 cases of uterine cavity adhesion, pelvic cavity infection 5 cases |  |  |
| Guo Yi-hong, 2010 | 200 (100 / 100) | ②④ | 6 | uterine cavity adhesion 6 cases | 17 | uterine cavity adhesion  17 cases |  |  |
| Zhuang Shui-lian, 2012 | 400 (200 / 200) | ②④ | 0 | menostasis 0 case | 9 | Menostasis 9 cases | 0 | 13 |
| Guo Yin-e, 2012 | 160 (80 / 80) | ②④ | 0 | menostasis 0 case | 21 | Menostasis 21 cases | 0 | 11 |
| Zhang Lin-lin, 2014 | 305 (150 / 105) | ②④ | 16 | Hypomenorrhea or menostasis 8 cases, uterine cavity adhesion 8 cases | 35 | Hypomenorrhea or menostasis1 8 cases, uterine cavity adhesion 17 cases |  |  |
| Wen Hua-ying, 2014 | 120 (60 / 60) | ②④ |  |  |  |  | 0 | 9 |
| Liu Jian-wen, 2010 | 119 (60 / 59) | ②④ | 1 | 1 case of pelvic cavity infection | 2 | 2 cases of pelvic cavity infection |  |  |
| Sun Yang, 2014 | 180 (90 / 90) | ②④ |  |  |  |  | 0 | 5 |
| Pan Cui-hong, 2013 | 162 (81 / 81) | ②④ |  |  |  |  |  |  |
| Xu Hong, 2014 | 260 (180 / 180) | ②④ |  |  |  |  | 0 | 2 |
| Zhan An-na, 2014 | 100 (50 / 50) | ②④ | 2 | uterine cavity adhesion 2 cases | 8 | uterine cavity adhesion 8 cases |  |  |
| Huang Li-guang, 2014 | 240 (120 / 120) | ②④ | 2 | menostasis 2 cases | 17 | menostasis 17 cases | 0 | 5 |
| Zong Ji-wei, 2013 | 100 (50 / 50) | ②④ | 0 | pelvic inflammation 0 case | 1 | pelvic inflammation 1 case | 0 | 10 |
| Han Ying, 2013 | 180 (90 / 90) | ②④ |  |  |  |  |  |  |
| Luo Ning, 2013 | 180 (90 / 90) | ②④ | 1 | menostasis 0 case, pelvic inflammation 1 case | 9 | menostasis 2 cases, pelvic inflammation 7 cases | 0 | 5 |
| Zhang Dong-ling, 2013 | 145 (70 / 70) | ②④ |  |  |  |  | 1 | 10 |
| Wang Ju-hui 2013 | 300 (150 / 150) | ②④ | 0 | menostasis 0 case, | 20 | menostasis20 cases, | 0 | 10 |
| Yao Wen-lei, 2013 | 152 (76 / 76) | ②④ | 1 | menostasis 1 case, | 3 | menostasis 3 cases |  |  |
| Lu Ling, 2013 | 210 (105 / 105) | ②④ | 0 | uterine cavity adhesion 0 case | 5 | uterine cavity adhesion 5 cases |  |  |
| Chen Yan-hong, 2013 | 178 (89 / 89) | ②④ | 2 | pelvic cavity infection 2 cases | 4 | pelvic cavity infection 4 cases |  |  |
| Li Ya-jun, 2012 | 120 (60 / 60) | ②④ | 2 | intrauterine adhesion 2 cases | 7 | intrauterine adhesion 7 cases |  |  |
| Shen Xiu-xiang, 2012 | 120 (60 / 60) | ②④ | 2 | pelvic cavity infection 2 cases | 8 | pelvic cavity infection 8 cases |  |  |
| Xiang Dong-shun, 2012 | 200 (100 / 100) | ②④ |  |  |  |  | 0 | 8 |
| Zhang Zhong-hua, 2012 | 224 (112 / 112) | ②④ |  |  |  |  | 0 | 4 |
| Chen Li, 2012 | 400 (200 / 200) | ②④ |  |  |  |  |  |  |
| Xu Yan-li, 2012 | 320 (160 / 160) | ②④ | 0 | menostasis 0 case, | 18 | menostasis 18 cases, | 0 | 9 |
| Zou Xia, 2012 | 200 (100 / 100) | ②④ | 0 | menostasis 0 case, | 8 | menostasis 8 cases, | 0 | 15 |
| Fan Jin-chan, 2012 | 140 (70 / 70) | ②④ |  |  |  |  | 0 | 4 |
| Xu Yun-e, 2012 | 240 (120 / 120) | ②④ |  |  |  |  | 0 | 6 |
| Yao Ling, 2011 | 210 (105 / 105) | ②④ | 0 | menostasis 0 case, uterine cavity adhesion 0 case | 17 | Menostasis 13 cases, uterine cavity adhesion 4 cases | 0 | 11 |
| Hou Lian-yun, 2011 | 160 (80 / 80) | ②④ |  |  |  |  | 0 | 6 |
| Lv Xiao-lan, 2011 | 100 (50 / 50) | ②④ | 0 | menostasis 0 case | 2 | menostasis 2 cases |  |  |
| Wu Cheng-zhen, 2011 | 400 (200 / 200) | ②④ | 1 | cervical adhesion 1 case | 4 | cervical adhesion 4 cases | 0 | 1 |
| Li Xin-ting, 2011 | 170 (85 / 85) | ②④ | 4 | uterine cavity adhesion 4 cases | 14 | uterine cavity adhesion  14 cases | 0 | 5 |
| Wang Wen-rong, 2011 | 240 (120 / 120) | ②④ | 0 | menostasis 0 case | 4 | menostasis 4 cases | 0 | 8 |
| Meng Xiao-yan, 2011 | 666 (320 / 346) | ②④ |  |  |  |  | 0 | 5 |
| Li Jian-xiu, 2010 | 128 (64 / 64) | ②④ | 4 | uterine cavity adhesion 4 cases | 13 | uterine cavity adhesion  13 cases |  |  |
| Hu Ting, 2009 | 260 (130 / 130) | ②④ | 0 | menostasis 0 case | 18 | menostasis 18 cases | 0 | 9 |
| Du Ai-qi,2009 | 550 (274 / 276) | ②④ | 4 | pelvic cavity infection 2 cases, intrauterine adhesion 2 cases | 9 | pelvic cavity infection  4 cases, intrauterine adhesion 5 cases |  |  |
| Zhao Cai-qin, 2011 | 2209110 / 110) | ②④ |  |  |  |  | 0 | 11 |
| Qin Li-na, 2012 | 300 (150 / 150) | ②④ | 2 | pelvic cavity infection 1 case, cervical adhesion 1 case | 5 | pelvic cavity infection  3 cases, cervical adhesion  2 cases | 0 | 6 |
| Guo Yu-sheng, 2013 | 100 (50 / 50) | ②④ | 4 | cervical adhesion 4 cases | 14 | cervical adhesion 14 cases |  |  |
| Wang Xu-yin, 2011 | 385 (185 / 200) | ②④ | 11 | cervical adhesion 11 cases | 34 | cervical adhesion 34 cases |  |  |
| Tang Yong-zhen, 2014 | 110 (55 / 55) | ②④ |  |  |  |  |  |  |
| Zeng Yin-ying, 2013 | 100 (50 / 50) | ②④ | 0 | No complications | 4 | pelvic cavity infection  2 cases, cervical adhesion  1 case, menostasis 1 case | 0 | 6 |
| Ou Ji-lan, 2014 | 200 (100 / 100) | ②④ | 0 | menostasis 0 case | 7 | menostasis 7 cases | 0 | 8 |
| Yang Yong, 2014 | 184 (94 / 90) | ②④ | 2 | cervical adhesion 2 cases | 9 | cervical adhesion 9 cases |  |  |
| Yu Chan-yang, 2014 | 160 (80 / 80) | ②④ |  |  |  |  | 0 | 9 |
| Zhang Yuan, 2014 | 240 (120 / 120) | ②④ | 0 | pelvic cavity infection 0 case | 8 | pelvic cavity infection 8 cases | 0 | 4 |
| Lei Xun, 2012 | 180 (90 / 90) | ②④ | 2 | pelvic cavity infection 1 case, uterine cavity adhesion 1 case | 7 | pelvic cavity infection  3 cases, uterine cavity adhesion 4 cases | 0 | 4 |
| Han Dong-hong, 2012 | 300 (150 / 150) | ②④ |  |  |  |  | 0 | 4 |
| Xie Feng,2009 | 379 (198 / 199) | ②④ |  |  |  |  |  |  |
| Chen Si-feng, 2013 | 240 (120 / 120) | ②④ | 2 | pelvic cavity infection 2 cases, menostasis 0 case | 18 | menostasis 6 cases, pelvic cavity infection 12 cases | 0 | 10 |
| Zhang Chang-qing, 2011 | 274 (149 / 98) | ②④ |  |  |  |  |  |  |
| Zhang Chun-yan, 2013 | 480 (240 / 240) | ②④ | 13 | 13 cases of uterine cavity adhesion | 50 | 50 cases of uterine cavity adhesion |  |  |
| Rui Hong-wei, 2013 | 632 (318 / 314) | ②④ | 0 | 0 case of pelvic cavity infection; 0 case of cervical adhesion; | 31 | 18 cases of pelvic cavity infection; 13 cases of cervical adhesion | 0 | 16 |
| Zhang Ying-hui, 2013 | 105 (50 / 55) | ②④ |  |  |  |  |  | 1 |
| Yan Wei-wei, 2013 | 320 (160 / 160) | ②④ | 0 | menostasis 0 case | 19 | menostasis 19 cases | 0 | 15 |
| Feng Ming-yue, 2012 | 160 (80 / 80) | ②④ |  |  |  |  |  |  |
| Sun Guang-fan, 2013 | 1000 (500 / 500) | ②④ | 0 | menostasis 0 case | 15 | menostasis 15 cases | 0 | 24 |
| Liu Jian-hua, 2014 | 208 (100 / 108) | ②④ |  |  |  |  |  | 3 |
| Huang Yin, 2014 | 136 (68 / 68) | ②④ |  |  |  |  |  |  |
| Wang Xue-yan, 2014 | 260 (130 / 130) | ②④ | 0 | cervical adhesion 0 case, menostasis 0 case, pelvic cavity infection 0 case | 6 | cervical adhesion 2 cases, menostasis 3 cases, pelvic cavity infection 1 case | 0 | 5 |
| Lan Chong, 2013 | 200 (100 / 100) | ②④ |  |  |  |  | 0 | 2 |
| Xia Li-hua, 2013 | 1186 (593 / 593) | ②④ |  |  |  |  | 0 | 29 |
| Ai Yin-hui, 2012 | 140 (70 / 70) | ②④ |  |  |  |  |  |  |
| Xue Lei, 2011 | 240 (120 / 120) | ②④ |  |  |  |  |  |  |
| Meng Xia, 2011 | 120 (60 / 60) | ②④ |  |  |  |  |  |  |
| Yi Jing-qiu, 2014 | 200 (100 / 100) | ②④ | 1 | pelvic inflammation 1 case, cervical adhesion 0 case | 12 | pelvic inflammation 8 cases, uterine cavity adhesion  4 cases | 0 | 7 |
| Zheng Chun-qin, 2014 | 198 (99 / 99) | ②④ |  |  |  |  | 0 | 8 |
| Liu Min, 2014 |  | ②④ |  |  |  |  |  |  |
| Zhang Li-rong, 2014 | 104 (52 / 52) | ②④ |  |  |  |  | 1 | 9 |
| Zhang Xin, 2014 | 420 (210 / 210) | ②④ | 6 | pelvic cavity infection 5 cases, uterine cavity adhesion 1 case | 19 | pelvic cavity infection  14 cases, uterine cavity adhesion 5 cases | 0 | 4 |
| Yang Ni, 2013 | 176 (88 / 88) | ②④ | 2 | pelvic inflammation diseases 2 cases | 6 | pelvic inflammation diseases 6 cases | 2 | 9 |
| Zhu Dan-yang, 2014. | 167 (85 / 82) | ②④ |  |  |  |  |  |  |
| Zhang Yu-rong, 2014 | 240 (120 / 120) | ②④ |  |  |  |  | 0 | 20 |
| Wu Dan-mei, 2010 | 200 (100 / 100) | ②④ |  |  |  |  | 0 | 3 |
| Lv Mei, 2013 | 200 (100 / 100) | ②④ |  |  |  |  | 0 | 4 |
| Zhang Yong-cun, 2013 | 502 (267 / 235) | ②④ |  |  |  |  | 3 | 47 |
| Zhang Fa-li, 2013 | 200 (100 / 100) | ①③ | 0 | pelvic cavity infection 0 case, uterine cavity adhesion 0 case | 0 | pelvic cavity infection 0 case, uterine cavity adhesion 0 case |  |  |

Medicine Taking Plan

①The patients in the research group take OC.

②The patients in the research group take OC and traditional Chinese medicine.

③The patients in the control group take placebo, blank control, condoms, and the couple follow other contraceptive measures.

④The patients in the control group orally take traditional Chinese medicine.
